# The Biological Clock in Gray Mouse Lemur: Adaptive, Evolutionary and Aging Considerations in an Emerging Non-human Primate Model

**DOI:** 10.3389/fphys.2019.01033

**Published:** 2019-08-09

**Authors:** Clara Hozer, Fabien Pifferi, Fabienne Aujard, Martine Perret

**Affiliations:** UMR CNRS MNHN 7179, Brunoy, France

**Keywords:** circadian clock, gray mouse lemur, aging, non-human primate model, adaptation, evolution

## Abstract

Circadian rhythms, which measure time on a scale of 24 h, are genetically generated by the circadian clock, which plays a crucial role in the regulation of almost every physiological and metabolic process in most organisms. This review gathers all the available information about the circadian clock in a small Malagasy primate, the gray mouse lemur (*Microcebus murinus*), and reports 30 years data from the historical colony at Brunoy (France). Although the mouse lemur has long been seen as a “primitive” species, its clock displays high phenotypic plasticity, allowing perfect adaptation of its biological rhythms to environmental challenges (seasonality, food availability). The alterations of the circadian timing system in *M. murinus* during aging show many similarities with those in human aging. Comparisons are drawn with other mammalian species (more specifically, with rodents, other non-human primates and humans) to demonstrate that the gray mouse lemur is a good complementary and alternative model for studying the circadian clock and, more broadly, brain aging and pathologies.

Back to the lemur: 30 years of chronobiology studies in a primate

“Time is an illusion.”

Albert Einstein

## Introduction

Circadian rhythms are biological rhythms that display an endogenous period of approximately 24 h. They are widely distributed in all living organisms, from cyanobacteria to mammals as well as plants (Loros and Dunlap, 2001; Ditty et al., 2003). They are the external expression of an internal biological clock driven by external environmental *stimuli*, chief among which is the cycle of days and nights induced by Earth rotation. Temperature, food availability and social interactions also influence endogenous clock expression (Dibner et al., 2010). The endogenous clock controls vital physiological, metabolic and behavioral processes such as hormone secretions, temperature, cellular metabolism and locomotor activity (LA) (Aschoff, 1983; Dvornyk et al., 2003; Lin et al., 2009; Bass and Takahashi, 2010). It synchronizes these functions to light-dark cycles to anticipate the environmental changes associated with the solar day. Moreover, it coordinates intrinsic activities with each other, suggesting a high adaptive value (Pittendrigh, 1993; Paranjpe et al., 2003; Sharma, 2003). This could explain why circadian clocks are ubiquitous in living organisms, even those living in constant darkness (e.g., in natural caves) (Koilraj et al., 2000) or artificially maintained in aperiodic environments (Sheeba and Sharma, 1999).

The circadian clock is composed of a central pacemaker located in the suprachiasmatic nuclei (SCN) of the anterior hypothalamus and peripheral oscillators in different organs (Buijs et al., 2013; Moore, 2013). Light entrainment of natural cycles requires retinal ganglion cells (RGCs) that contain melanopsin and are intrinsically photosensitive (Rollag et al., 2003; Hankins et al., 2008). Melanopsin-expressing RGCs are considered the main mediator of circadian photoentrainment and directly transmit information to the SCN via nervous pathways, which thereby synchronizes all peripheral clocks via hormones such as melatonin or corticosterone (Yamaguchi et al., 2003). Cones and rods also contribute to encoding light intensity for photic entrainment (Dkhissi-Benyahya et al., 2007; Güler et al., 2008).

Without any environmental cues, the circadian clock still displays endogenous periodicity close to 24 h in most animal species (Pittendrigh and Daan, 1976a), which is referred to as the free-running period, or *tau* (Pittendrigh, 1960; Halberg, 1962), and is generated and sustained intracellularly by a transcription-translation negative feedback loop involving several genes (e.g., *Clock, Per* or *Bmal1*) (Gekakis et al., 1998; Yu et al., 2002; Duong et al., 2011; Lande-Diner et al., 2013). It has been assumed that fitness is enhanced when the endogenous clock closely matches environmental periodicity (Pittendrigh and Daan, 1976a). When reared under light-dark cycles that deviated from 24 h (21 or 27 h), fruit flies (*D. melanogaster*) exhibit a significantly shorter lifespan than flies under 24 h cycles (Pittendrigh and Minis, 1972). This study first introduced the “circadian resonance hypothesis,” stating that eukaryotic systems perform most effectively as oscillators when they are driven close to their natural “circadian” frequency. Wyse et al. (2010) found that in several mammal species, deviation of *tau* from 24 h is inversely related to longevity, which supports Pittendrigh’s hypothesis.

During aging, changes in the circadian rhythmicity of endocrine, metabolic and behavioral properties have been described in several mammalian species, characterized by amplitude alterations, phase delays or period modifications, revealing potential internal desynchronization (Turek et al., 1995; Valentinuzzi et al., 1997; Weinert, 2000; Kondratova and Kondratov, 2012). In rodents, age-related wheel-running activities are characterized by an increase in these rhythmic deteriorations, which might be related to anatomical and functional declines within the SCN (Farajnia et al., 2012; Nakamura et al., 2015, 2016). Indeed, age-related circadian changes may be related to lower sensitivity to light of the circadian system (Witting et al., 1993), though the underlying mechanisms remain unknown for the moment.

**FIGURE 1:**
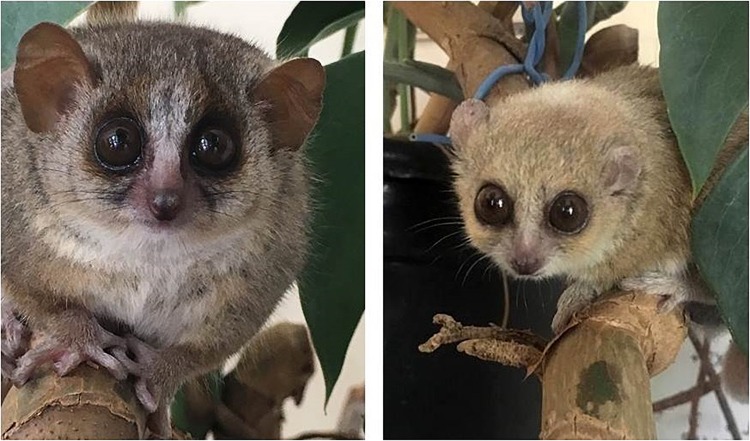
One-year old female mouse lemur (*M. murinus*) **(left)** and 7-year old male mouse lemur **(right)** in the breeding colony of Brunoy. Credit: Aude Noiret.

In the present review, we focus on the gray mouse lemur (*Microcebus murinus*, Figure 1), a Malagasy non-human primate belonging to the suborder Strepsirhini and to the Cheirogaleidae family, which includes small, omnivorous primates. Gray mouse lemurs are nocturnal, solitary foragers and sleep in groups during the daytime. In its natural environment, the gray mouse lemur faces dramatic seasonal environmental variations. During the hot rainy season (from October to March), characterized by a long photoperiod, elevated temperatures and abundant food resources, the mouse lemur exhibits a high level of activity, a high metabolic rate during the daily dark phase and mating behavior. Conversely, the cooler dry season (from April to September) is characterized by harsh conditions in terms of food resources or temperature. These seasonal changes represent a challenge in that it is necessary to adapt biological rhythms and energy expenditures. At the onset of the dry season (photoperiod shorter than 12 h), mouse lemur metabolism dramatically slows down, leading to an increase in fat deposits and the occurrence of pronounced daily phases of hypometabolism (Schmid and Speakman, 2000; Génin and Perret, 2003). These physiological changes are strictly dependent on photoperiod (Génin and Perret, 2000; Perret and Aujard, 2001b). In captivity, gray mouse lemurs reach ages of up to 12 years (Languille et al., 2012). In the wild, their life span is significantly lower (Lutermann et al., 2006). The half-life is generally used to delineate adults from aged individuals and is approximately 5–6 years in captive mouse lemurs (Perret, 1997; Pifferi et al., 2018). Both physiological and behavioral parameters show a decrease after 5.5 years (Aujard and Perret, 1998; Némoz-Bertholet and Aujard, 2003), allowing the discrimination of young and aged animals.

Although this species is nocturnal, *M. murinus* is a convenient model for studying chronobiology because its behaviors, biological rhythms and physiological functions depend strongly on photoperiod. In addition, due to exhibiting a relatively small body size (head-body length of approximately 15 cm) and low body mass (60–80 g), mouse lemurs can be easily bred and kept in captivity. This makes them an ideal laboratory model among non-human primates, offering a compromise between practical breeding methods and physiological and phylogenetic proximity to humans. Finally, mouse lemurs have increasingly gained attention as a promising model for human aging (Bons et al., 2006; Languille et al., 2012; Picq et al., 2015), particularly in the context of research on neurodegenerative diseases or age-related perturbations of biological rhythms in humans. Indeed, they develop cerebral age-related impairments (in cognitive flexibility for instance) similar to those found in aged humans, as recently illustrated by Joly et al. (2014) and Picq et al. (2015). These deteriorations include circadian rhythm alterations such as progressive fragmentation of LA during life (Aujard et al., 2006).

Since the early 1970s (Martin, 1972), seasonal and daily rhythms have been studied, and this review includes published data on circadian rhythms in mouse lemurs. Long seen as an “archaic” or basal primate, the gray mouse lemur is actually fully adapted to its fluctuating environment, particularly in terms of circadian rhythms. This review aims to demonstrate that *M. murinus* may be regarded as a promising aging circadian model for humans. The first part details the mouse lemur’s circadian clock characteristics and daily entrainment, which is followed by a description of the environmental factors affecting them. The third part investigates the effect of age on the circadian clock. Finally, evolutionary considerations about the mouse lemur’s circadian characteristics close this review. An additional table drawing up a comparison of gray mouse lemur, rodents, human and other primate species’ circadian features, is available in Supplementary Table S1.

## Daily Entrainment of the Circadian Clock by Light and its Characteristics

**FIGURE 2:**
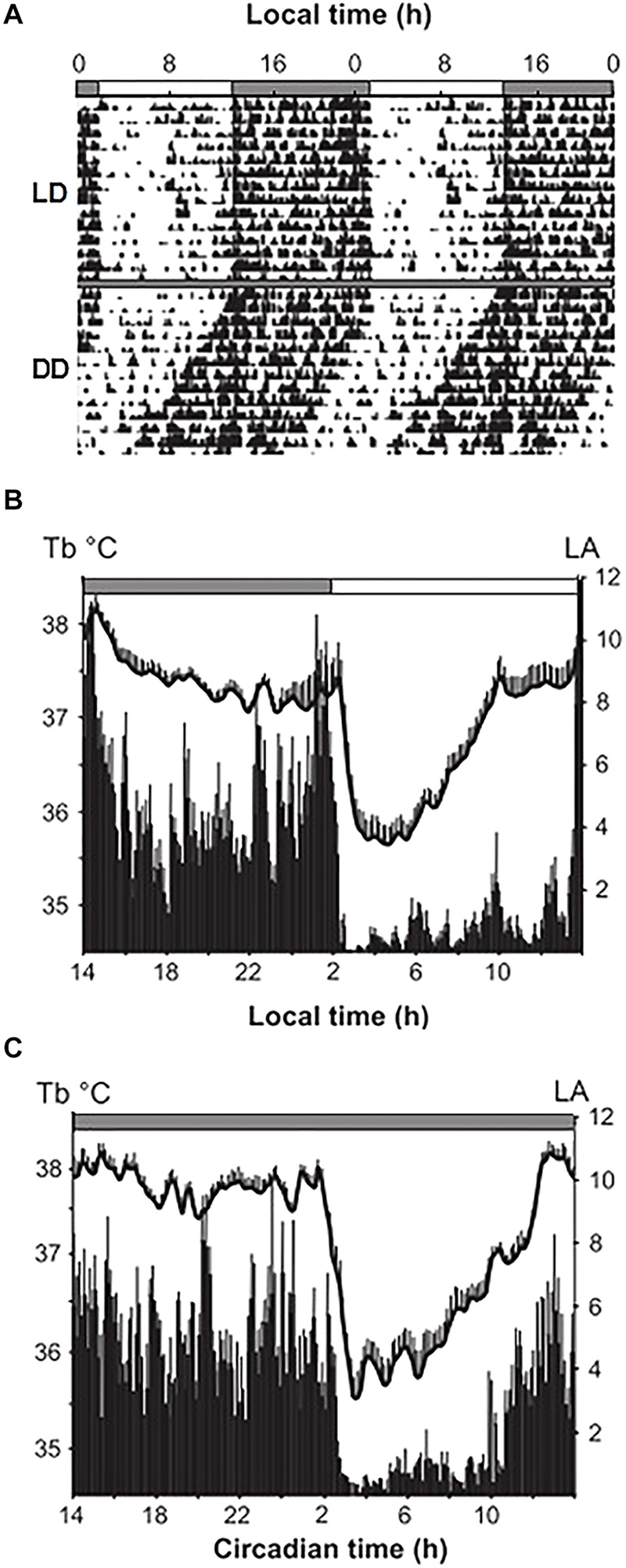
Representative outputs of locomotor activity (LA, arbitrary units) and body temperature (Tb, C) rhythms in mouse lemurs exposed to control light-dark cycles (LD, 12:12, night at 14 h, thick gray bar on top) and animals under free-running conditions (DD, continuous dark indicated by a thin gray bar). **(A)** Actogram (double plotted) over a 30 day-period under LD and DD. **(B)** (Mean SEM) Tb (black curve) and LA rhythm (histograms) profiles over a 7-day period under LD and **(C)** under DD conditions (from Perret et al., 2010).

Under constant conditions of ambient temperature and a 12:12 h light-dark cycle, the gray mouse lemur exhibits a robust circadian rhythm typical of a nocturnal species (Figure 2). LA is strictly restricted to the dark phase and is associated with high body temperature (Tb). During the light phase, mouse lemurs exhibit daily hypothermia phases lasting several hours, starting with a linear and rapid drop in Tb, leading to minimal Tb after 3 h on average (Perret and Aujard, 2001a).

To assess changes in circadian rhythms in animals exposed to variations in environmental conditions, two parameters were considered as important markers: body temperature and LA.

### General Characteristics of the Endogenous Biological Clock

#### Endogenous Circadian Characteristics

In an aperiodic environment without any light or social cues, the endogenous period (also called *tau* or the free-running period) is one of the main characteristics of the biological clock and represents the duration of a complete circadian cycle (Aschoff, 1960). This cycle can be divided into the subjective night and the subjective day, which correspond to the active and resting phases of an individual, respectively. When mouse lemurs are kept under constant darkness (free-running DD – Figure 2) and constant ambient temperature, LA and body temperature exhibit strong circadian periodicity typical of a nocturnal species, with high levels of LA and a higher Tb during subjective night (Perret and Aujard, 2001a). As found in many nocturnal species (Pittendrigh and Daan, 1976a), the gray mouse lemur clock oscillates with a period of less than 24 h: on average 23.6 + 0.2 h (Aujard et al., 2006). When maintained under constant light (free-running LL), light exerts a strong inhibitory effect on LA in the strictly nocturnal gray mouse lemur, but constant conditions do not prevent temperature from exhibiting circadian rhythms.

As in all small mammals, daily hypothermia occurring under free-running conditions (either DD or LL) is a component of the circadian organization of the mouse lemur and cannot be manifested without functional circadian system. Indeed, bilateral lesions of the SCN affect the expression of natural daily hypothermia in Djungarian hamsters (*Phodopus sungorus*) (Ruby et al., 1989). In a wide range of endotherms using daily hypothermia and living in seasonally predictable climatic conditions, such as the mouse lemur, daily hypothermia is expressed predominantly during the cold season and during reproduction in summer (Kenagy, 1989; Körtner and Geiser, 2000; Schmid and Speakman, 2000; Génin and Perret, 2003). The annual alternation of reproductive phase and sexual rest associated with hypothermia is controlled by the photoperiod via the pineal gland. In laboratory conditions, Djungarian hamsters exposed to a short photoperiod exhibit testicular regression followed by the expression of daily hypothermia (Heldmaier and Steinlechner, 1981).

#### Phase-Response Curve

**FIGURE 3:**
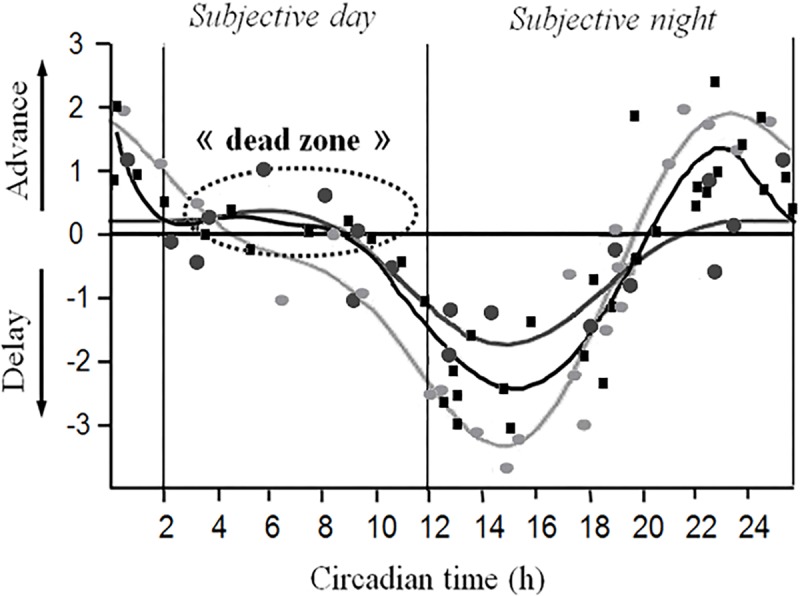
Superposition of the phase-response curves (PRC) of mouse lemur obtained after 1 h bright white light pulses (black line and squares) and human subjects after 1 h (dark gray line and circles) and 6.7 h (light gray line and circles) bright white light pulses maintained under free-running dark-dark conditions. The raw data (symbols) as well as three-harmonic fits (continuous lines) are represented (from Schilling et al., 2001; St Hilaire et al., 2012). The mouse lemur PRC falls between the two human PRCs. Note the absence of a “dead zone” in the 6.7 h human PRC.

In nocturnal species, such as the gray mouse lemur, light drives entry into the resting phase. To determine the phase-response curve (PRC), individuals under free-running DD were subjected to 1 h light pulses. These light pulses may have different effects: phase advance, phase delay, i.e., shifts in the onset of activity in the next cycle according to the circadian time of delivery or no effect at all (Pittendrigh and Daan, 1976b). The PRC was established using 49 young and old individuals (Figure 3). All animals exhibited fast resynchronization independent of age. A greater amplitude of phase delay (−2 h to −3 h) than phase advance (+1 h) was also observed, revealing a “dead zone” in the PRC (Schilling et al., 2001). According to a general rule described by Pittendrigh and Daan (1976b, c), individuals with a short *tau* should exhibit greater delay and less advancement, which was obviously the case with the gray mouse lemur.

Despite a general pattern of the PRC that is similar in gray mouse lemurs and rodents, nocturnal and diurnal rodent species exhibit phase delays almost twice as long as those of the gray mouse lemurs, as observed in the golden Hamster (Pohl, 1984) or in mice and rats (Pittendrigh and Daan, 1976b). This characteristic may be a typical primate trait, since similar observations have been made in other primate species, such as owl monkeys (*Aotus lemurinus*) (Rauth-Widmann et al., 1991), marmosets (*Callithrix jacchus*) (Wechselberger and Erkert, 1994) or squirrel monkeys (*Saimiri sciureus*) (Hoban and Sulzman, 1985).

In humans, highly comparable characteristics of the PRC are observed when the temperature rhythm (Minors et al., 1991) or plasma or salivary melatonin (St Hilaire et al., 2012; see Figure 3) is used as a circadian marker under different light pulse durations. Minors et al. (1991) subjected volunteers to 3 h pulses, whereas St Hilaire et al. (2012) compared 1 h and 6.7 h pulses, with different but consistent results (Figure 3). When subjected to 1 h pulses, human individuals display shorter advances and delays (maximum −1.75 h and +0.45 h) than when subjected to 6.7 h pulses (maximum −3.44 h and +2.02 h) but longer delays than when subjected to 3 h pulses (maximum ∼2 h of advance and delay). As the PRC of the mouse lemur was established only using 1 h light pulses, its shape cannot be predicted under 7 h light pulses. The human PRC displays a lower amplitude of phase delays and advances under the same duration of light pulses compared to the mouse lemur species, whose PRC is located halfway between the human and rodent phase-resetting responses.

### Daily Entrainment by Light and Limits of the Response

#### Resynchronization After a Phase Delay or Advance

The gray mouse lemur displays a capacity for quick synchronization and resynchronization of its rest-activity circadian rhythms. Within a few minutes after lights are turned off, the animals become active, regardless of their age (Schilling et al., 2001). When subjected to a 6-h advance or delay, mouse lemurs synchronize their LA and body temperature rhythms within 2 or 3 days (Schilling et al., 2001; Perret et al., 2003). These findings contrast with the 8 days required for synchronization after an 8 h delay (3.7 days after a phase advance) recorded by Schanz et al. (1987). Moreover, the shorter the *tau*, the quicker resynchronization to a 6-h phase advance is (Perret et al., 2003).

In rodents, a greater number of transient cycles is required to adjust activity rhythms to shifts, especially in old individuals (between 4 and 5 days, Halberg, 1969; Valentinuzzi et al., 1997). In other primates, studies show divergent results: the galago (*Otolemur garnettii)*, a nocturnal prosimian, requires from 10 to 12 days for reentrainment (Erkert et al., 2006) to an 8-h advance or delay, whereas the Senegal bushbaby (*Galago senegalensis*) requires approximately 9 or 5 days, respectively (Schanz and Erkert, 1987). The human situation is more questionable. Buresova et al. (1991) showed that early morning bright light advances the human circadian melatonin concentration within 1 day, whereas other studies report a synchronization delay of approximately 3–4 days (Piérard et al., 2001; Czeisler et al., 2016). The fast resynchronization of the gray mouse lemur could therefore be a typical adaptation of this species.

#### Limits of Synchronization Determined by Changing Night Length

**FIGURE 4:**
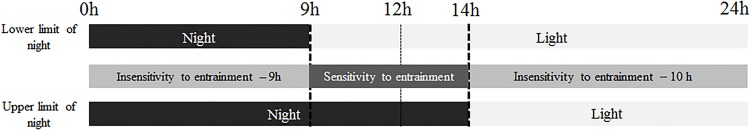
Limits of synchronization determined by changing the night duration per 24 h in the mouse lemur. To be synchronized, the night duration must last at least 9 h and must not exceed the duration of nighttime (14 h). Beyond these limits, temperature and locomotor activity desynchronize.

Entrainment by light occurs within precise limits owing to the presence of phases of insensitivity to light. To characterize the limits of entrainment, 48 males were exposed to nighttime durations ranging from 16 h per day to 5 h per day as well as free-running DD and free-running LL. The markers of the rhythms were Tb, LA and daily hypothermia. Whatever the nighttime duration, LA was strictly restricted to dark phases, with a total masking effect of light during the light phase. Focusing on LA during dark phases, the reduction of the nighttime duration was not compensated by an increase in LA during the dark phase. With respect to body temperatures, daily hypothermia was directly induced by light only if night duration did not go beyond the length of subjective night (around 14 h). However, in animals exposed to 24-h light-dark cycles with nighttime ranging from 10 to 5 h, the lower limit of the induction of daily hypothermia by light was 9 h of night (Perret and Aujard, 2001a). Beyond these limits, temperature and LA desynchronize in relation to phases of insensitivity to light (Figure 4).

These precise limits of synchronization are due to phases of insensitivity to light, as found in other mammals, even though their limit of responsiveness seems to be much wider. For example, two species of mammals, the flying squirrel (*Glaucomys sabrinus*) and the chipmunk (*Tamias striatus*), are capable of achieving synchronization when the photoperiod is less than 3 h per 24 h day and longer than 18 h, with relatively stable phase angles (DeCoursey, 1972). Furthermore, LA and plasma melatonin rhythms remain synchronized to the light-dark cycle in all photoperiods in Soay sheep (from 8 h to 22 h light per day) (Wagner et al., 2008).

In conclusion, the differences expressed in terms of reentrainment to a phase advance or delay and the limits of light entrainment show great variability within mammalian species. Varied responses do not seem to be particularly related to the respective circadian periods (*tau* < / > 24 h) but are the expression of high phenotypic flexibility. Circadian clocks therefore cannot be seen as fixed systems imposed on a certain temporal niche but, rather, can be seen as plastic structures whose behavioral outputs adapt optimally to external environmental conditions, according to ecological constraints to perfectly match their circadian characteristics with various abiotic and biotic environmental parameters.

### Mouse Lemur Daily Sleep Rhythms

To our knowledge, only two studies have investigated the sleep-wake pattern of the gray mouse lemur based on electroencephalographic (EEG) rhythms. Adult mouse lemurs exposed to long daylengths exhibit approximately 55% wakefulness and 45% sleep over a 24 h period. Sleep generally occurs during the diurnal resting phase (near 71% of the recording time), whereas activity represents almost 90% of the total nocturnal active phase (Pifferi et al., 2012). The rhesus monkey (*M. mulatta*) displays a very similar sleep-wake distribution during daytime (Hsieh et al., 2008), but the two species differ on two points. First, rhesus monkeys spend more time sleeping (89%) during the rest phase than mouse lemurs (71%). This divergence could be associated with the different durations of the rest phases in each species (8 h per day in rhesus monkeys versus 14 h per day in mouse lemurs in winter). Second, mouse lemurs exhibit a greater number of sleep bouts, which reflects a much more fragmented sleep pattern during the rest phase than that of rhesus monkeys, which exhibit a consolidated sleep period of approximately 8 h, similar to humans, with only brief arousals throughout the nighttime. This trait is comparable to the fragmented sleep pattern found in rodents: in constant conditions, rats sleep between 30 and 40% of the subjective night (activity phase) and display more than 100 sleep–wake transitions in the course of the circadian cycle (Mistlberger et al., 1983; Tobler, 1995; Revel et al., 2009). Sleep fragmentation is typical of small vertebrates and may be due to energetic constraints (Capellini et al., 2008a; Roth et al., 2010). However, mouse lemurs usually sleep in groups (Perret, 1998); the isolated conditions of the tested individuals during the experimentation might have changed their sleep-wake sequences and could have a significant effect on rhythm fragmentation, which should be investigated.

Despite its rodent-like fragmented sleep pattern, the mouse lemur exhibits deep slow–wave sleep (SWS) that is much closer to that in humans than that in rodents. This sleep phase (often referred to as deep sleep) is marked by slow, high-amplitude EEG waves. Rodents display a higher frequency of SWS (9–13 Hz, SWS1, Panagiotou et al., 2017) than humans (1–3 Hz, SWS4, McCarley, 2004), whereas the mouse lemur’s SW lies in-between (1–8 Hz, SWS2, Pifferi et al., 2012; Royo et al., unpublished). The rat and the mouse lemur share a similar brain size (Le Gros Clark, 1931), but mouse lemurs have larger neocortices that are composed of more cortical areas and expanded parietal and temporal regions. In fact, a greater number of neocortical areas is a characteristic of primates in general, particularly in parietal, temporal, and frontal regions, which are proportionally larger in primates (Halley and Krubitzer, 2019). These characteristics might explain why sleep patterns in mouse lemurs are similar to those in other small mammals in their structure but are similar to those in other primates in their electrophysiologic characteristics. All these observations make the mouse lemur an interesting intermediate sleep model between rodents and humans.

### Underlying Mechanisms of Clock Photic Entrainment

#### Evening and Morning Oscillators

Since light-dark cycles have been identified as the strongest environmental cues, wavelength and light intensity obviously play a major role in the clock synchronization of mammals (Berson et al., 2002; Berson, 2003; Duffy and Wright, 2005). The circadian clock, located in the SCN, receives environmental inputs via photopigments located in the eyes and from melanopsin-expressing RGCs. It has also been suggested that the onset and cessation of animal activity are controlled by two coupled oscillators (evening and morning receptors). A variation in wavelength or light intensity may therefore influence the rest-activity pattern of individuals. In non-human primates, only a small amount of information about circadian photoentrainment is available. In some lemurs, it has been suggested that cones play a role in circadian light perception (Deegan and Jacobs, 1996; Dkhissi-Benyahya et al., 2001; Erkert et al., 2006). In the gray mouse lemur, synchronization increases with the light intensity and is better for mid-wavelengths (470–540 nm) than for short and long wavelengths (Perret et al., 2010). In most organisms, twilight transitions are the dominant environmental stimuli involved in synchronization of the circadian phase (Boulos et al., 2002). For mouse lemurs, the most efficient wavelengths evoke synchronization at light intensities ranging from 0.5 to 1 lux (a threshold that is among the lowest in nocturnal species (Erkert, 2008), which are comparable to dawn and dusk light, respectively, in Malagasy forest, when dominant wavelengths are shifted toward mid-wavelengths of 450–500 nm (Pariente, 1980). Synchronization also appeared to be more efficient for dark-light transitions than for light-dark transitions, probably due to the greater sensitivity of photoreceptors to light, leading to a better response in the SCN (Boulos et al., 1996; Comas et al., 2008; Refinetti, 2008).

#### Olfactory Bulb Influence

Olfactory bulbs seem to play a major role in the expression of biological rhythms. In rodents, removal of olfactory bulbs affects diverse outputs of daily or seasonal rhythmicity, such as the rest-activity pattern, changes in light-dark activity ratios, lengthening of the circadian period, alterations in activity amplitude, etc. (Possidente et al., 1990, 1996; Vinkers et al., 2009). In the mouse lemur, removal of the olfactory bulbs alters the gonadal responses to photoperiod, with a delay in testis development and reduced testosterone levels, which highlights the role of olfactory bulbs in the neuroendocrinological control of seasonal rhythmicity (Perret and Schilling, 1993; Schilling and Perret, 1993). More seasonal responses of the energy balance are modified in bulbectomized animals (Séguy and Perret, 2005a). On a daily scale, bulbectomized males in free-running DD displayed a significantly shorter circadian period of body temperature and LA (22.4 ± 0.2 h vs. 23.4 ± 0.3 h for control males). Although bulbectomized males exhibited robust circadian rhythms, they showed a delay in entry into daily hypothermia and increased diurnal activity bouts (Perret et al., 2003). The effect of olfactory bulb activity on the circadian clock could be mediated by social olfactory cues (Goel and Lee, 1997; Granados-Fuentes et al., 2006), or even by light, since it has been proven that PER1 and PER2 gene expression in olfactory bulbs is light-sensitive in mice (Hamada et al., 2011). All these effects are not directly related to the sense of smell but, rather, are due to the neural projections from olfactory bulbs to the SCN (Granados-Fuentes et al., 2006). Besides, the analysis of the sensorial pathway in gray mouse lemur has shown efferent projections of the olfactory bulbs on the SCN, among other brain regions, exhibiting a direct link between the olfactory system and the central pacemaker (Mestre et al., 1992). Nevertheless, the underlying mechanisms of the precise role of olfactory bulbs in endogenous clock resynchronization remain largely unknown.

#### Cellular Aspects

The SCN are located in the hypothalamus and receive inputs from light via the retinohypothalamic tract that reaches the ventral part of the SCN, where specific neuron populations that communicate via peptide and protein secretions are located (Moore and Speh, 2004). Among these neurons, the vasoactive intestinal polypeptide (VIP)-containing neurons, confined within the ventral region, are directly influenced by photic input from the retina in rodents and in humans (Mai et al., 1991; Moore and Danchenko, 2002). Their daily VIP levels remain constant under constant light conditions in rats, whereas they show a daily rhythm under LD conditions, underlying the important involvement of this peptidergic cell population in circadian rhythm control (Ibata et al., 1989; Shinohara et al., 1993; Dardente et al., 2004). The VIP neurons project to the dorsal part (or shell) of the SCN, where neurons containing arginine vasopressin (AVP) settle, which are one of the largest neuron population of the SCN (Moore and Danchenko, 2002) and are implicated in the coupling of SCN neurons (Li et al., 2009; Yamaguchi et al., 2013). These two neuronal populations are therefore deeply involved in circadian rhythmicity control, spreading photic information to other neural target sites, leading to specific rhythm expression, such as sleep/wake cycles (Kalsbeek et al., 2010; Jones et al., 2018).

In the mouse lemur, few studies have addressed cellular aspects of the biological clock. However, daily fluctuations in AVP and VIP neurons have been characterized. These neurons are located in the dorsal and ventral parts of the SCN, as found in rodents and humans (van Esseveldt et al., 2000). VIP neurons are exclusively found in the core SCN lying adjacent to the optic chiasma. However, AVP neurons, although mainly present in the shell SCN, are located outside the SCN as well, in the supraoptic nucleus, medial preoptic area, and the bed nucleus of the stria terminalis, as found in rodents (Dong and Swanson, 2006; Xu et al., 2018). VIP neurons are smaller than AVP neurons. Both neuron types exhibit strong daily rhythms under 14:10 h LD cycles, showing inverse activity throughout the day: AVP neurons display their highest and lowest activity and intensity levels during daytime and nighttime, respectively (with a peak at the end of daytime and a drop at the beginning of nighttime); VIP neurons behave in the opposite manner (Cayetanot et al., 2005a; Aujard et al., 2006).

Being a nocturnal species does not prevent the mouse lemur from displaying similar synchronization mechanisms to humans. Indeed, most genetic and physiological circadian parameters are nearly identical in diurnal and nocturnal species, showing specific temporal expression over a 24 h day (Challet, 2007). The expression of melatonin, for instance, is restricted to nighttime and is mainly driven by the circadian clock in both diurnal and nocturnal species (Simonneaux and Ribelayga, 2003; Emet et al., 2016; Touitou et al., 2017). In both categories of animals, light pulses at night activate Fos expression and induce the expression of the Per1 and Per2 mRNA and proteins in the SCN (Rose et al., 1999; Yan et al., 1999; Dkhissi-Benyahya et al., 2000; Dardente et al., 2002; Yan and Okamura, 2002; Caldelas et al., 2003; Matìjù et al., 2010; Schwartz et al., 2010; Grone et al., 2011; Morin, 2013). Anatomically, Per1 and Per2 are highly expressed in AVP-containing neurons (dorsomedial part of the SCN) but are expressed at lower levels in the VIP-containing neurons (ventrolateral part of the SCN); this segregation is found in both diurnal and nocturnal species (Dardente et al., 2002; Yan and Okamura, 2002; Hamada et al., 2004; Foley et al., 2011; Vandunk et al., 2011). To sustain circadian signals intracellularly, the negative-feedback loops between Clock/Bmal1 heterodimers and Per and Cry gene transcription are thought to be identical in diurnal and nocturnal mammals (Yu et al., 2002; Duong et al., 2011; Lande-Diner et al., 2013; Bollinger and Schibler, 2014). In addition, studies conducted in night-active and day-active mammalian species reveal that the phase-shifting effect of light is mainly efficient during the night period, regardless of the chronotype (Pohl, 1984; Takahashi et al., 1984; Hoban and Sulzman, 1985; Slotten et al., 2005; Shuboni and Yan, 2010). In the daytime, light exerts no synchronization effect during the previously described dead zone of the PRC, located between two windows of responsiveness to illumination. Therefore, under laboratory conditions, underlying photic resetting is closely similar in nocturnal and diurnal mammals. This suggests that differences between the two groups may lie in mechanisms downstream of the SCN pacemaker, certainly at the hormonal, glucose or lipid control level (Kumar et al., 2015).

## Influence of Environmental Factors on Mouse Lemur Circadian Rhythms

Circadian rhythms are not static processes and are subject to environmental influences, similar to other physiological and metabolic parameters (Rand et al., 2006; Ciarleglio et al., 2011; Azzi et al., 2014; van der Vinne et al., 2014; Riede et al., 2017). In the mouse lemur, some physiological constants, such as temperature, daily hypothermia or LA, may be affected by environmental changes. In this section, an attempt is made to draw up a list of several abiotic (temperature, food availability, light intensity) and biotic (social interactions) parameters that influence the daily pattern of circadian rhythms in the gray mouse lemur and demonstrate the plasticity of their expression.

### Abiotic Influence on the Daily Pattern of Temperature and Locomotor Activity

Mouse lemurs experience large variations in environmental conditions (Song and Geiser, 1997; Withers et al., 2000). During the cold and dry winter season, resources are limited, which contrasts with the breeding season during the hot summer season. In response to external conditions, mouse lemurs counteract environmental challenges by adjusting their energy expenditures through daily modifications of their internal body temperature, mainly via hypothermia expression and LA. As mentioned above, these mechanisms are controlled by the circadian clock (Perret and Aujard, 2001a). The diurnal decrease in body temperature is an important adaptive energy-saving strategy that is adjusted to ecological constraints and controlled by the biological clock (Aujard and Vasseur, 2001; Giroud et al., 2008; Terrien et al., 2009).

#### Effect of Ambient Temperature

In response to both daily and seasonal changes in ambient temperature (T_*a*_), mouse lemurs adjust their energy expenditure through daily hypothermia. Experimental exposure to T_*a*_ ranging from 20 to 12°C increases the duration and depth of hypothermia bouts in association with a large decrease in the minimal Tb (Séguy and Perret, 2005b; Terrien et al., 2009). Hypothermia responses to cold T_*a*_ are regularly observed during the winter season and remain unusual during the breeding season. Indeed, in the mouse lemur, daily hypothermia during the reproductive season never results in substantial energy benefits and can have potential adverse consequences (body mass loss, oxidative stress, DNA damage, sleep deficit, etc., Giroud et al., 2008; Villain et al., 2016). While hypothermia and reproduction may be mutually exclusive in the mouse lemur, as in most rodents, this does not apply for all mammalian species. Echidnas, marsupials, and some other placentals resort to hypothermia during the reproductive season, although hormonal and energetic features seem incompatible. In the wild, similar results have demonstrated the high flexibility of daily hypothermia in the mouse lemur. A minimal Tb of 7.7°C and hypothermia bouts of up to 22 h have been recorded in wild animals during the winter season (Schmid and Speakman, 2000; Dausmann, 2014). Finally, when exposed to hot ambient temperatures (>28°C), mouse lemurs do not display daily hypothermia (Aujard et al., 1998). In natural conditions, mouse lemurs avoid high summer T_*a*_ by resting in buffered cavities (Goodman et al., 1993; Radespiel et al., 2003; Lutermann et al., 2010).

Low ambient temperature and body energy reserves and food scarcity are known to be the main trigger of daily hypothermia. Facing low food availability and/or T_a_, mouse lemur require larger thermoregulatory investments, which usually considerably enhances hypothermia expression prevalence (Séguy and Perret, 2005b). However, the daily profile of Tb during hypothermia and the time of arousal, appear to be more fixed and controlled largely by the circadian clock.

The effects of T_a_ on other circadian aspects have not yet been studied in the mouse lemur, but they have been examined in other primates such as southern pig-tailed macaques (*Macaca nemestrina*), squirrel monkeys, marmosets and owl monkeys, which are four New-World monkey species. Variations in T_a_ do not lead to major effects on individuals’ circadian characteristics (with no effect on *tau*, T_a_ acts as a weak synchronizer, since it fails to entrain free-running activity and Tb rhythms in the owl monkey under trapezoidal T_a_ cycles of 20-30°C, Erkert et al., 2006). In all studied species, a lower T_a_ causes an increase in activity, but a decrease in Tb, even though activity and Tb patterns as well as *tau* remain unchanged. In some individuals, however, the circadian Tb rhythm also shows pronounced short-term variations, exhibiting an earlier or delayed Tb drop under cold exposure, manifesting in the existence of ultradian modulations at the Tb level (Erkert, 2008), as found in mouse lemurs subjected to a cold environment (Terrien et al., 2009). In humans, cold or heat exposure affects the Tb pattern and sleep structure: heat exposure increases wakefulness and thermal load during sleep and decreases SWS and rapid-eye-movement (REM) sleep; cold exposure mainly affects REM sleep due to the suppression of the thermoregulatory response (Muzet et al., 1984; Okamoto-Mizuno and Mizuno, 2012).

#### Effect of Light Intensity

In addition to changes in photoperiod that have clear effects on circadian rhythm patterns, changes in light intensity during the night or light phase can also influence these patterns. Marked species-specific differences exist in the circadian system’s susceptibility to entrainment to light intensity. An intensity of 0.1 lux (full-moon luminosity, Kyba et al., 2017) during the light phase is sufficient to entrain mouse lemur and owl monkey circadian activity to 24 h (Erkert and Thiemann, 1983; Perret et al., 2010). By contrast, this intensity fails to entrain the endogenous rhythm of the Senegal bushbaby and the galago, whose threshold for photic entrainment lies at approximately 3–30 lux (Erkert et al., 2006). These observations highlight the differences in the circadian system’s threshold for photic entrainment, even within nocturnal prosimians.

Mouse lemurs maintained under first in free-running DD, then either under dim light (DimL, 50 Lux, 628 nmol photons/s/m^2^) or under free-running LL (150 lux, 2600 nmol/s/m^2^) showed significant elongation of *tau* between free-running DD, DimL and LL (from 23.3 ± 0.5 h in DD to 24.3 ± 0.2 h in DimL, and from 23.1.0 ± 0.2 h to 25.4 ± 0.2 h in LL). LA was also negatively affected by DimL and constant light, but DimL still allows quite normal LA, whereas constant light exerts a masking effect on LA (Le Tallec, 2015, Figure 5). Nevertheless, dim light has been shown to decrease melatonin levels in mouse lemurs (Le Tallec et al., 2016). Indeed, when exposed to 51.5 lux dim light, mimicking streetlight pollution, during their active dark phase, mouse lemurs display a significant drop of urinary 6-sulfatoxymelatonin concentrations related to alterations of daily rhythm profiles compared to mouse lemurs subjected to 0.1 lux full-moon-simulating light. These results suggest that light at night confuses day length perception and affects proper photoentrainment. These results corroborate Aschoff’s rule (Aschoff, 1960), which originally stated that in nocturnal animals, *tau* is positively correlated with light intensity, whereas the inverse correlation is observed in diurnal animals. Although this rule is currently challenged in diurnal species (especially in mammals, among which humans, whose free-running period lengthens with increasing illumination (Wever, 1989), it remains mostly true in nocturnal mammals, birds, fishes and reptiles (Aschoff, 1979).

**FIGURE 5:**
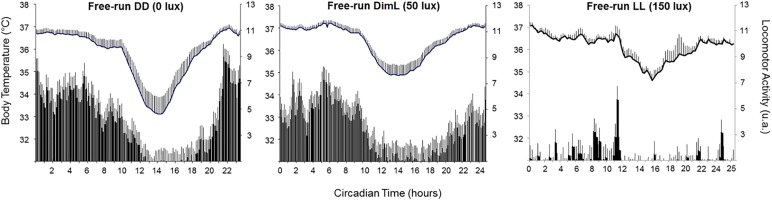
Body temperature and locomotor activity of 26 male mouse lemurs under free-running dark-dark (DD), dim-light (DimL), and light-light (LL).

The high threshold for photic entrainment in some species may be due to an adaptive character preventing the endogenous clock from being affected by moonlight. Surprisingly, the mouse lemur does not display such high photic threshold of entrainment, and one may wonder whether moonlight affects the mouse lemur’s biological rhythms or not and to what extent it contributes to its environment adaptation. Light is known to exert a strong masking effect on mouse lemurs (which is also observed in the owl monkey, Erkert and Gröber, 1986; Fernández-Duque et al., 2010), and full-moon luminosity can prevent mouse lemurs from leaving their nest and, thus, being seen and preyed upon by owls, snakes and other carnivorous nocturnal mammals.

#### Effect of Nutrition on Circadian and Daily Rhythms

The circadian timing must synchronize environmental cues to ensure maximum performance at a given time (Hasegawa and Arita, 2014). The SCN drives activity/sleep rhythms that determine feeding hours and metabolic activity, which are strong entraining signals. A good illustration of the strong power of the circadian clock in modulating metabolic rhythms is food anticipatory activity, reflected by increases in locomotion, corticosterone secretion, body temperature and several metabolic outputs before food distribution in rodents (Mistlberger, 1994; Stephan, 2002). Conversely, feeding behavior and food intake can impact circadian clock outputs. The effects of nutrition on circadian rhythms have been largely documented (Mendoza, 2007). When animals are submitted to precise daily feeding schedules, they synchronize partially or completely a wide variety of their biological rhythms. Mice show a peak of activity in their lateral and ventromedial hypothalamic nuclei entrained to the time of feeding (Kurumiya and Kawamura, 1991). Likewise, a shift in the expression of *Per1* and *Per2* occurs in the cerebral cortex with peaks at mealtime, differing from the nocturnal peak in animals fed *ad libitum* (Wakamatsu et al., 2001).

##### Food intake

In the gray mouse lemur, a chronic food shortage of 80% in summer season leads to advancement of entry into hypothermia by 16 ± 6 min from the 16th day and increases in the length of hypothermia bouts of 9 ± 5 min/day during the first 25 days of restriction. These effects are greater under short days (winter season) since either 40 or 80% caloric restriction advances the entry into hypothermia by 10 ± 3 min/day and increases hypothermia bouts by 30 ± 6 min/day during the first 14 days (Giroud et al., 2008, 2009). In winter, the gray mouse lemur displays a phenotype reflecting its behavioral and sexual inactivity. Therefore, adjusting early its body temperature enables quick energy savings, besides an autumnal fattening in order to cope with seasonal lack of food (Perret, 1998). These responses are greatly enhanced when restriction of food intake is associated with low ambient temperature (Séguy and Perret, 2005b). Finally, nest sharing by mouse lemurs may counteract the effects of cold exposure and/or food restriction on daily T_a_ and LA (Séguy and Perret, 2005b).

##### Nutrients

Relationships between dietary manipulation and patterns of Tb and LA rhythms have been tested in mouse lemurs. One study reported that resveratrol dietary supplementation significantly shortens the free-running period in both young and old animals: 23.15 ± 0.09 h vs. 22.90 ± 0.12 h in supplemented young animals, 23.00 ± 0.10 h vs. 22.49 ± 0.14 h in supplemented old animals (Pifferi et al., 2011a). This effect is supported by previous findings in rat fibroblast cells in which the expression of circadian clock genes had been modified by resveratrol (Oike and Kobori, 2008). A potential mechanism would involve resveratrol-induced activation of the SIRT1 gene, whose activity is closely linked to *Clock* and *Bmal1* activity (Nakahata et al., 2008). This property could be relevant in the context of some circadian clock disruption pathologies. A second study showed that resveratrol supplementation improved synchronization with the light-dark cycle, inducing a reduction of LA onset and a delay of the time from which mean Tb starts to decrease, leading to diminution of the hypothermia duration (Pifferi et al., 2013). Finally, polyunsaturated fatty acids (PUFAs) have been shown to influence daily patterns of Tb, especially the implementation of daily hypothermia. n-3 PUFA supplementation reduces the depth and length of daily hypothermia (Vuarin et al., 2016).

### Social Interactions Influence Circadian Rhythms

Light is known to be the main *zeitgeber* to synchronize the circadian clock with environmental cues. However, social interactions can also be powerful *stimuli* to reset circadian rhythms, by affecting the light input and the pattern of light exposure, adjusting the period of circadian clock (shown in humans, Mistlberger and Skene, 2005). In several primate species, social synchronization of activities within a group is generally observed (Erkert et al., 1986; Erkert and Schardt, 1991; Melo et al., 2013). To determine whether social interactions may affect the periodicity of circadian rhythms in the members of a group, 12 male mouse lemurs were tested to determine their free-running period for 15 days, then were paired for 10 days and then returned to isolation for 5 days in darkness (Séguy, 2005).

**FIGURE 6:**
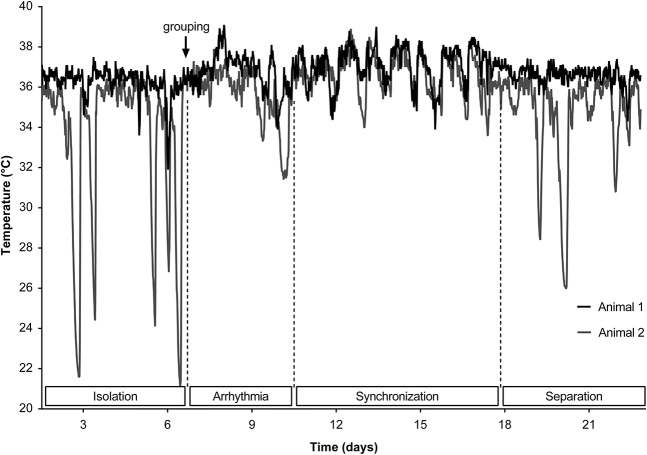
Synchronization of Tb free-running rhythms in two male individuals: phase of arrhythmia 3–4 days after grouping, followed by synchronization characterized by the same free-running period length, and then desynchronization when the animals are isolated again.

Once paired, 4 of the 6 groups synchronized their rhythms after 6 ± 0.4 days, manifested by the absence of a phase difference between the two individuals, sometimes after a phase of total asynchrony (Figure 6). Two groups did not exhibit synchronization before the end of the experiment. This result is similar to results found in palm squirrel and marmosets (Erkert and Schardt, 1991; Rajaratnam and Redman, 1999). The lack of convincing evidence of entrainment for two individuals may lie in the difference in *tau* before pairing or the timing of the endogenous cycle in which the animals were paired. Hence, the existence of a “sensitivity window,” i.e., a precise time range in the endogenous cycle that enables social entrainment, might be assumed. Another reason may lie in individual differences in the sensitivity of the circadian system to social cues (Rajaratnam and Redman, 1999).

Further investigations were conducted to determine the nature of the entraining signal. Visual and olfactory contacts appear to be strong synchronizers (Séguy, 2005). Chemical signals that are known to interfere in social relationships between individuals (reproduction, individual recognition, social organization, etc.) in mouse lemurs and other mammalian species (Doty, 1976; Perret, 1986; Braune et al., 2005; Wolff and Sherman, 2007; Tobin et al., 2010; Aglioti and Pazzaglia, 2011; Kulahci et al., 2014; Polese et al., 2015) can therefore also affect circadian synchronization (Erkert and Schardt, 1991; Goel and Lee, 1996; Mistlberger and Skene, 2004; Silva et al., 2005; Favreau et al., 2009).

## Evolution of the Circadian Pacemaker Over the Lifetime: Effect of Age on the Expression of Biological Rhythms

In non-human primates, knowledge about rhythmic perturbations during aging remains scarce and mainly comes from rhesus monkeys and mouse lemurs, whose longevities in captivity are 35–40 (Bodkin et al., 2003) and 10–12 years (Languille et al., 2012), respectively. It is generally agreed that age-related changes observed in biological rhythms are caused by the deterioration of the time-keeping system (Hofman, 2000; VanSomeren, 2000; Hofman and Swaab, 2006). These alterations can concern the circadian clock itself or associated physiological and behavioral processes, such as the activity-rest rhythm or temperature patterns. The study of the effects of aging on the characteristics of the endogenous clock and its capacity to respond to light entrainment is particularly pertinent in the gray mouse lemur, which has a longer life span than rodents and is less subject to social bias than humans and other social primates.

### Changes in *tau* With Age

The relationship between aging and the endogenous period in the gray mouse lemur has been investigated several times, but different studies have led to contradictory conclusions. A longitudinal study performed by Schilling et al. (2001) on four individuals showed no age-related changes of *tau*, except that one animal displayed an increase in *tau* with age. By contrast, two transverse studies comparing young (2–4.5 years) and old (5–9 years) animals found a significant decrease in *tau* between the two groups, with an average 0.75 ± 0.15 h decrease (Cayetanot et al., 2005b; Aujard et al., 2006). Such divergent conclusions may lie in the differences in the experimental design of either longitudinal (Schilling et al., 2001) or transverse studies (Cayetanot et al., 2005b; Aujard et al., 2006). Moreover, the number of individuals varies from one study to another, as does the photoperiodic regimen. Further longitudinal studies are required to conclude definitely on this issue, since the evolution of *tau* seems to be highly individual-dependent.

It is even more difficult to determine the age-related effects on *tau* when they actually differ from one species to another. In rodents, it is commonly accepted that *tau* becomes shorter with age (Pittendrigh and Daan, 1976a; Moore-Ede et al., 1982), but some studies indicate that *tau* remains stable in Syrian hamster (Davis and Viswanathan, 1992). In humans, the same uncertainties persist: Weitzman et al. (1982) then Monk and Moline (1989) showed an effective decrease in temperature *tau* with age in subjects from 20 to 80 years, but Czeisler et al. (1999) revealed that *tau* of young and old individuals were closely comparable, at approximately 24.18 h, as did Kendall et al. (2001). This finding could support the hypothesis that different underlying mechanisms control the expression of *tau*. Finally, aging can be regarded as an individual process that leads to great interindividual variations, preventing the assessment of a clear effect at the global level. In Schilling et al. (2001), study among the 4 old animals that survived the longitudinal experiment, two individuals maintained stable circadian rhythms and died at older ages, whereas the two others displayed a shortening of *tau* and died soon after the experiment. This observation could reveal that lifespan may depend on the homeostasis of biological constants that differ considerably from one individual to another, making it difficult to generalize the impact of aging on the expression of the biological clock and its underlying mechanisms.

The evolution of *tau* during life raises the question of the adaptive impact of aging on an individual’s fitness. For example, several studies have found that a diverging *tau* relative to 24 h was linked with a decreased lifespan (Pittendrigh and Minis, 1972; Hurd and Ralph, 1998; Wyse and Coogan, 2010; Gutman et al., 2011; Libert et al., 2012). First described by Pittendrigh and Minis (1972), this theory, known as the theory of circadian resonance, states that the organism fits perfectly its environment when its circadian system oscillates with a period of 24 h (i.e., the circadian system, “resonates”), which enhances individual survival and lifespan. According to this assumption, organisms with a free-running period far from 24 h require daily synchronization to external environmental cycles resulting in a physiological cost corresponding to the deviation of *tau* from 24h, which may impact fitness. In this context, a divergent *tau* at an advanced age can quickly become deleterious by disturbing the proper entrainment of the circadian master clock. Due to a loss of normal phase-relationships between the endogenous oscillator and the fixed environmental period, desynchronization of behaviors such as diurnal activity or chronic phase advances would decrease fitness and, in turn, survival in aged mouse lemurs.

### Effects of Age on Daily and Seasonal Rhythms of LA and Tb

Compared to adult animals, aged mouse lemurs display a decrease in LA amplitude, an advanced activity onset and an increase in daytime activity associated with fragmentation (Cayetanot et al., 2005b; Aujard et al., 2006). However, although these results have been observed in humans as well as other non-human primates and rodents (Vitiello et al., 1986; Czeisler et al., 1992; Valentinuzzi et al., 1997; Weinert et al., 2000; Weinert and Waterhouse, 2007; Zhdanova et al., 2011; Duffy et al., 2015), they do not explain whether these observed age-related alterations are due to a reduction of sensitivity to external light factors or to changes within the clock mechanism itself. However, a high incidence of ocular pathologies has been identified in mouse lemurs that are older than 7 years (Beltran et al., 2007; Alleaume et al., 2017; Dubicanac et al., 2017). This strongly suggests a decrease in light responsiveness through filtering of short wavelengths that are known to be efficient in the synchronization of daily rhythms in mouse lemurs (Gomez et al., 2012).

Aging is also associated with immune system alterations in the gray mouse lemur. Indeed, plasma levels of interferon-γ (IFN-γ, a cytokine regulating immune and inflammatory responses intervening in the pathogenesis of a number of brain diseases, Blasko et al., 2001) are correlated with age-related disturbance of circadian rhythms and survival: high levels of IFN-γ are associated with a short lifespan and a short free-running period *tau*; IFN-γ levels also correlate with characteristic patterns of LA and body temperature during aging (high percentage of diurnal LA, advanced onset, delayed occurrence of minimal Tc, Cayetanot et al., 2009).

Perturbations of Tb rhythm are particularly impacted with age because of the modification of the daily pattern of hypothermia. During aging, diurnal hypothermia actually tends to disappear, consequently reducing the Tb amplitude (Perret and Aujard, 2006). These modifications of LA and Tb rhythms are related to other age-related energetic and hormonal rhythm perturbations that are expressed seasonally.

Seasonal alternations are characterized by changes in daylength and temperature, among other factors, which are of major relevance to the expression of activity patterns and reproductive function in primates (Chik et al., 1992; Hill et al., 2004a, b). With age, the synchronization of circadian rhythms is altered in regard to daylength: a decrease in the sensitivity of the circadian clock to short-term light-dark cycles modification (Zhang et al., 1996; Benloucif et al., 1997) and age-related modulation of the temporal organization of daily rhythms (Scarbrough et al., 1997; Benstaali et al., 2002; Martin et al., 2003).

With aging, mouse lemurs display a decrease in the amplitude of the seasonal variations in body mass, basal metabolic rate, sexual hormones, and DHEA-S (Aujard and Perret, 1998; Aujard et al., 2001; Perret and Aujard, 2005). In response to exposure to a long photoperiod, old mouse lemurs show an increase in interdaily variability and a decrease in the amplitude of LA with a phase advance compared to younger animals (Aujard et al., 2007), providing evidence of impairment of mechanisms involved in both light perception and SCN activity.

### Changes in Sleep During Aging

Aging has been associated with numerous and diverse changes in sleep. In humans, these changes include an increase sleep fragmentation, decreases in total sleep time, sleep efficiency and SWS, and attenuation of EEG slow-wave activity (SWA, EEG power density between 0.75–4 Hz) in NREM sleep (Landolt et al., 1996; Carrier et al., 2002; Crowley, 2011; Luca et al., 2015). The age-related alterations of sleep-wake rhythms in the mouse lemur consist of decreased activity (−20%) during the active phase, more active wakefulness (+50%) and a reduction in SWS (−40%) during the resting phase (Pifferi et al., 2011b). Comparable observations have been made in other mammals, such as humans. Aged rhesus monkeys, for example, display reduced daily activity duration, as well as high day-to-day variability in sleep quantity and quality. This is associated with fragmentation of LA during nighttime and daytime, with more sleep during daytime, and shortened time spent in REM sleep and SWS (Zhdanova et al., 2011). The results in rodents are more contradictory. Old mice exhibit more sleep and more SWS and non-rapid eye movement sleep (light sleep) during the resting phase, characterized by an increased amplitude and steeper slopes, which surprisingly is the opposite of what is found in humans and other non-human primates (Panagiotou et al., 2017). However, several studies in rats show that, despite more desynchronized sleep and sleep bouts, neither active wakefulness nor SWS is altered during aging (Zepelin et al., 1972), whereas others have shown a significant increase in the time spent awake and a decrease in active sleep time (van Gool et al., 1987). Mouse lemurs also display chronic phase advances, resulting in an earlier wake-up in the morning, as observed in older humans and rodents (Yunis et al., 1974; Duffy et al., 1998; Monk, 2005). In this regard, the mouse lemur exhibits age-related sleep-wake alterations similar to those found in humans and can therefore be seen as a compelling aging model of sleep rhythm disturbances.

### Underlying Mechanisms of the Aging of the Circadian Clock

Age-related changes in the circadian clock are linked with anatomical and practical disruptions of the SCN (Antle and Silver, 2005; Nakamura et al., 2011). Despite some studies in rodents, the underlying mechanisms of the alterations of the biological clock with age remain unclear. However, a decrease in sensitivity to light demonstrated by reduced Fos expression in the SCN has been described in aging rodents (Sutin et al., 1993; Benloucif et al., 1997). Alterations of neurochemical and electrophysiological aspects of the SCN have also been reported, including changes in the VIP and AVP expression (Roozendaal et al., 1987; Kawakami et al., 1997; Kalló et al., 2004), reduced amplitude of electrical activity rhythm (Nygård et al., 2005; Biello, 2009), altered melatonin production with age and recovery of young-like expression of certain clock genes upon melatonin administration in rats (Manikonda and Jagota, 2012; Mattam and Jagota, 2014). Modification of the ability of the SCN to reset peripheral clocks can be mentioned as well when rats are subjected to a 6-h advance or delay (Davidson et al., 2008). The most conclusive evidence of the decisive role of the SCN in aging is the restoration of some of these alterations after transplantation of the SCN from fetal to aged individuals (Viswanathan and Davis, 1995; Cai et al., 1997).

In mouse lemurs, young and old animals exhibit significant differences in urinary sulfatoxymelatonin (aMT6s). During the night period, the urinary aMT6s values of young individuals increase immediately after the onset of darkness (from 40 ng/mg Cr to 120 ng/mg Cr), whereas the urinary aMT6s of old individuals remain low, near 30 ng/mg CR throughout the day/night period (Aujard et al., 2001). Using the early Fos gene response in the SCN to a light stimulus under different irradiance levels, the density of Fos induction in the SCN has been demonstrated to increase proportionately with increasing irradiance in young mouse lemurs. By contrast, exposure to low levels of irradiance fails to increase SCN Fos expression in aged individuals. Moreover, under an identical level of irradiance, Fos expression shows a reduction of 88% in aged mouse lemurs compared to young ones (Aujard et al., 2001). Finally, a decrease in Fos expression in the main olfactory bulbs following an odorant stimulus has been described in aged mouse lemurs, potentially explaining both the age-related decreases in behaviors associated with olfaction and indirect effects on SCN (Cayetanot et al., 2005b).

Changes in AVP and VIP have also been reported (Cayetanot et al., 2005a; Aujard et al., 2006). Although the number of AVP-positive neurons counted and the amplitude of their rhythm are comparable in adult and aged animals, the daily oscillation of this parameter is affected by aging, with a delay of the peak in the number of AVP neurons by approximately 4 h with respect to that in young animals. A similar pattern is found regarding VIP-positive SCN neurons, whose peak also shifts by 4 h in aged mouse lemurs. The presence of calcium-binding protein calbindin-D28K (CalB) cells in the SCN was also revealed in the gray mouse lemur. These CalB cells in the mid-causal region of the SCN of hamsters express the Fos protein in response to light pulses (Silver et al., 1996) and are related to AVP and VIP cells (LeSauter et al., 2002). In young mouse lemurs, nuclear CalB immunoreactivity displays large daily variations, ranging from 31.7 ± 4.0% of cells with immunopositive nuclei during daytime to 9.3 ± 2.8% during nighttime. Such variations are significantly reduced with aging (Cayetanot et al., 2009).

In summary, longitudinal and transverse assessments of circadian behavioral, physiological and cellular alterations have revealed that mouse lemurs exhibit many similar characteristics with human aging. These observations suggest that the mouse lemur can be considered as an ideal system to explore the mechanisms underlying the evolution of the circadian clock aging, whether it is healthy or pathological. For instance, healthy aging is associated with some physiological and behavioral changes occurring alongside in the same age scope (IFN-γ levels and fragmentation of LA patterns for example, Cayetanot et al., 2009). Further longitudinal studies should be useful for determining the dynamic evolution of circadian aging parameters and identifying predictive biomarkers of longevity and neuropathological aging.

## Evolutionary Considerations: Is the Mouse Lemur a Primitive Circadian Model?

A persistent view of primatology history indicates that lemurs, especially mouse lemurs, have conserved some physiological and behavioral characteristics of early primates, including small size, nocturnal behavior, a frugivorous-insectivorous diet, a solitary way of life, and altricial young individuals carried by the mouth (Szalay, 1968; Charles-Dominique and Martin, 1970; Cartmill, 1972, 1974; Eisenberg, 1981; Rutberg, 1983; Teaford et al., 1996; Ross, 2001; Strait, 2001). However, some studies have demonstrated that most of these characteristics evolved recently and appear to derive from a reduction in body size only 30 MA ago, deconstructing the myth of the primitive mouse lemur (Gebo, 2004; Génin and Masters, 2011). Regarding circadian characteristics, it seems that the mouse lemur’s characteristics are highly adaptive and do not necessarily come from an ancestral fixed state.

### Mouse Lemur Nocturnality: An Ancestral Trait?

There is much debate regarding whether mouse lemur nocturnality can be seen as an ancestral or evolved trait. Some evidence supports the view that nocturnal activity in Strepsirrhini occurs as an ancestral character in comparison to other primate suborders. Indeed, the primate ancestors (like most mammalians ancestors) are assumed to have been nocturnal, with primate diurnality appearing in the most recent common ancestor of the suborder Haplorrihini during the Mesozoic (Bininda-Emonds et al., 2007; Joffe et al., 2014). However, this hypothesis of a unique nocturnal ancestor has been recently challenged by several observations. First, studies on strepsirrhinian eye structure and opsin genes encoding retinal pigments have led to contradictory conclusions. The *tapetum lucidum*, a reflective structure located behind the retina of many nocturnal animals, is found in several diurnal lemuriform species (*Lemur catta*, Indridae) but not in *Eulemur* species, which are cathemeral (Peichl et al., 2017). Furthermore, nocturnal cheirogaleids (close cousins of mouse lemurs) possess alleles for trichromatic diurnal vision, although they are mainly dichromatic, which suggests the recent occurrence of nocturnality in this group (Tan and Li, 1999). Trichromatic vision has also been detected in the nocturnal folivorous wooly lemurs (genus *Avahi*) (Veilleux et al., 2014). Another study on 14 representative prosimian species provided an explanation for the color-sensitive photoreceptor opsin gene patterns among prosimians that suggests early loss of the middle-wavelength and long wavelength opsin gene polymorphism, indicating an early convergent shift from a diurnal to a nocturnal lifestyle in prosimians and, thus, in the mouse lemur (Tan et al., 2005). Second, reconstruction of the activity pattern of the fossil omomyiform *Teilhardina asiatica* and the visual system of adapiforms, two major Paleocene members of the earliest known primates (Gingerich, 1976; Martin, 1993; Silcox et al., 2007), corroborate the diurnal ancestor hypothesis. Observations of the *T. asiatica s*kull orbits combined with faunivory and phylogenetically based on character analysis of activity patterns provide support for the diurnality hypothesis (Heesy, 2009). Ankel-Simons and Rasmussen (2008) questioned the common assumption that ancestral primates were nocturnal on the basis of reviewing studies focusing on the morphology and physiology of the primate visual system (eye size, corneal size, retinal morphology, and opsin distribution). They found that Paleocene plesiadapiforms and Eocene euprimates fossils observation supports the hypothesis of both diurnality and nocturnality in early primates, without concluding with a clear statement. Thus, the traditional view of the mouse lemur’s archaic nocturnal character seems erroneous, or at least uncertain. The preceding observations tend to agree with the hypothesis of several convergent shifts to nocturnality in prosimian primates. Finally, Ankel-Simons and Rasmussen (2008) reviewed the morphological and physiological characteristics of the primate visual system and stressed how rapidly and readily diurnality may have switched to nocturnality and vice versa, highlighting the significant recent evolutionary flexibility in the visual system of primate lineages. In light of these statements, one might assume that analogies between diurnal and nocturnal species can be easily and appropriately drawn in terms of behavioral and physiological circadian outputs, without misunderstanding either chronotype, since molecular clock mechanisms must have remained highly similar in both diurnal and nocturnal primates.

### Highly Adaptive Daily Hypothermia Stemming From Convergent Evolution

Since the basal metabolic rate is inversely correlated with body mass in endotherms, important energetic demands and costs are extremely marked in small eutherians (Gillooly et al., 2001; Glazier, 2018). That is the reason why a lot of small species use adaptive physiological mechanisms to reduce their energy consumption during inactive times of the day (Martin and Yoder, 2014). Employing hypometabolism to lower Tb periodically can save considerable amounts of energy, since thermoregulation represents an important part of the daily energy allocation. The use of hypothermia in many small mammals and birds corresponds to a more apparent decrease in Tb, occurring mostly during the resting phase of the individual (Lyman et al., 1982; Ruf and Geiser, 2015). The mouse lemur’s daily hypothermia is unique among primates, being restricted to the Cheirogaleidae, and is highly comparable to that found in other small daily heterotherms (Lovegrove and Raman, 1998; Körtner and Geiser, 2000). As in all small heterothermic mammals, daily hypothermia is part of the normal circadian organization of the mouse lemur, but some external parameters (food availability, ambient temperature, body energy reserves) can trigger the timing of hypothermia onset, which varies considerably, since the primary role of hypothermia turns out to be energy conservation (Terrien et al., 2009; Vuarin et al., 2013; Faherty et al., 2017). Although the daily hypothermia of small eutherians was long regarded as a “primitive” or even “imperfect” (McNab, 1978), it is currently thought to be highly adaptive (Aujard et al., 1998; Génin and Perret, 2003). Two conflicting hypotheses suggest two different origins of mouse lemur daily hypothermia (Blanco et al., 2018). One states that ancestral strepsirrhinians were heterothermic, contributing to their survival during their oceanic trip to Madagascar. Cheirogaleids are suggested to have conserved this trait during their evolution, whereas other lemurs have lost it (Martin, 1993; Kappeler, 2000; Nowack and Dausmann, 2015). Another scenario assumes that ancestral cheirogaleids experienced a dwarfism episode during their evolution, representing a *de facto* acceleration of their metabolic rates (Génin and Masters, 2016). Therefore, daily hypothermia would have helped to cope with a more challenging environment and would have been derived from numerous independent convergent evolutionary events in cold or arid regions (Dausmann and Warnecke, 2016). Furthermore, one study suggests that early primates colonizing Madagascar were large-sized and rules out the extensive use of heterothermy by adapiforms (Masters et al., 2007). Finally, the discovery of an active heating process, non-shivering thermogenesis, using brown adipose tissue containing a protein called UCP (Uncoupling protein) and the original repartitioning of brown adipose tissue in the mouse lemur confirm the convergent evolution hypothesis (Génin et al., 2003).

### Ecology and Evolution of the Mouse Lemur Sleep Pattern

The mouse lemur displays a very polyphasic sleep pattern close to that of rodents. This characteristic, which is typical of small vertebrates, must be due to energetic constraints, rather than predation threats (Capellini et al., 2008b; Roth et al., 2010). Indeed, fragmented sleep is present in small body-sized species that display reduced sleep cycles: because of the more frequent need to feed, small species are not able to consolidate sleep into one unique bout as in other larger species. The shorter sleep duration in monophasic animals leads to believe that one daily bout sleep may consolidate sleep benefits in a more efficient way. Monophasic sleep is thought to be an evolved trait, since there is 99% support for polyphasic sleep as an ancestral character state (Capellini et al., 2008b). However, despite its rodent-like polyphasic sleep pattern, the mouse lemur exhibits a total sleep duration much closer to that of humans than that of rodents. Indeed, the total sleep duration, which is strongly influenced by phylogeny (Capellini et al., 2008a), is significantly shorter in the mouse lemur (approximately 10 h per day, Pifferi et al., 2012) than in most rodents (Campbell and Tobler, 1984). The case of the mouse lemur is therefore paradoxical because it displays both polyphasic sleep and a reduced sleep time, which can be detrimental in terms of sleep consolidation. We hypothesize that the short sleep duration of the mouse lemur is offset by SWS that is deeper than in rodents and close to that of humans. This sleep phase (often referred to as deep sleep) is marked by slow, high-amplitude EEG waves and may provide the cognitive consolidation needed to counteract the fragmented short sleep duration. Despite showing a brain size similar to rats (Le Gros Clark, 1931), mouse lemurs exhibit a greater number of neocortical areas, which is a characteristic of primates in general, particularly in the parietal, temporal, and frontal regions (Halley and Krubitzer, 2019). These characteristics might explain why sleep patterns in mouse lemurs are similar to those in other small mammals in their structure but are similar to those in other primates in their electrophysiological characteristics, reflecting the phylogenetic proximity of the mouse lemur to other primates. All these observations make the mouse lemur an interesting intermediate sleep model between rodents and humans.

## Conclusion and Perspectives

(1) The mouse lemur displays flexible biological rhythms, allowing it to fully adapt to its changing environment. This plastic phenotypic trait facilitates adaptation to unpredictable environmental seasonal variations in Madagascar. The mouse lemur expresses two completely opposite seasonal phenotypes that are a particularity of *Cheirogaleus* and *Microcebus*, two Malagasy cheirogaleid primates, and are only determined by photoperiod length and driven by the plastic biological clock (Kobbe et al., 2011). The daily hypothermia exhibited by the mouse lemur is also highly adaptive and flexible: it enables the lemurs to respond to a predictable cold environment or food shortage, leading to strategic minimized energy expenditure during the rest phase of the day. The temporal organization of daily hypothermia in the gray mouse lemur is regulated by endogenous circadian function and triggered by environmental conditions. Daily hypothermia is then an adaptive endogenous process presumed to have evolved in order to cope with great variability of seasonal resources, such that the favorable phenotype would be expressed at the appropriate time of year. In addition, an influence of the light phase duration on circadian characteristics, particularly on *tau*, is suspected in the mouse lemur. Indeed, Pittendrigh and Daan (1976a) demonstrated an influence of photoperiod duration on *tau* in several mammalian and bird species, which can be seen as an influence of season. The effect of 24 h days with short photoperiods in winter is evidently to bring a short *tau* closer to 24 h, but that trend is reversed in midsummer, when the after effect of the long photoperiod is to shorten *tau* again. Their interpretation tends toward lability of the pacemaker to minimize the “trauma” of daily phase-shift variation with the season. This hypothesis has not yet been verified in the mouse lemur, and further investigations on the topic are planned to bring new insights into the seasonal plasticity of the mouse lemur clock.

(2) The mouse lemur can no longer be seen as a primitive ancestral species but, rather, should be seen as a primate intermediate species between humans and rodents and as a functional analog in terms of biological rhythms. Several arguments support this point of view. First, the mouse lemur’s nocturnal behavior, long considered as an ancestral trait (due to the first mammals’ supposed nocturnality), is now believed, owing to morphological, anatomical and genetic analysis, to have resulted from a convergent shift from diurnality to nocturnality during the last 30 million years, fitting with the mouse lemur’s anti-predatory behavior (Génin and Masters, 2011). Second, the daily hypothermia expressed by the mouse lemur appears to be a highly adaptive behavior to save substantial amounts of energy that was derived from numerous convergent shifts (Blanco et al., 2018). Finally, the sleep structure of the mouse lemur falls between that of humans and rodents. Despite their rodent-like fragmented sleep pattern, mouse lemurs exhibit SWS that is much closer to that of humans than that of rodents, with a low frequency of SW, comparable to the human frequency (Pifferi et al., 2012). Thus, the gray mouse lemur, by displaying biological similarities with rodents while retaining some primate characteristics provides a noteworthy intermediate study organism.

(3) From a medical perspective, the mouse lemur is a very good model in terms of circadian clock characteristics during its life and aging in numerous regards, including sleep structure, changes in LA amplitude, increased daytime activity, advanced activity onset and the underlying cellular mechanisms during aging, etc. All these parameters are very similar in mouse lemurs and humans, except the mouse lemur’s daily hypothermia that can be seen as a specific trait and also as a limit for the mouse lemur as an animal model. Nevertheless, the additional fact that the mouse lemur displays spontaneous brain pathologies such as the formation of amyloid plaques that resemble those of Alzheimer’s disease, show that this species is currently seen as a new promising model for studying circadian disruptions and, more generally, cerebral pathologies in aging humans (Bons et al., 2006; Languille et al., 2012). Furthermore, its small size and weight make it an easy species to breed in laboratory conditions. However, the primate status of the mouse lemur can be constraining in some regards (ethical rules among other considerations), and it therefore cannot be used as a substitute for rodent species but as a complementary model (Fischer and Austad, 2011; Ezran et al., 2017).

(4) Despite some molecular studies on the mouse lemur’s biological clock, much remains to be discovered and described in this area, especially concerning the mechanisms underlying the alteration of the biological clock during aging, in contrast to studies on rodents, in which much more information is available. Nevertheless, the recently completed sequence-based *M. murinus* genome provides new tools and positive perspectives for measuring the genetic expression of circadian genes, among others, in this species (Larsen et al., 2017; Roberts, 2019).

(5) An additional table summarizing gray mouse lemur’s circadian characteristics in comparison with rodents, human and other primate species is available in the Supplementary Table S1. It highlights the domains or key questions that remain to be explored regarding the mouse lemur’s circadian clock such as the cellular and molecular mechanisms underlying mouse lemur’s clock, in particular during aging. Longitudinal study of the circadian clock would help clarifying the evolution of circadian constants during aging as well as the study of olfactory bulbs influence and their underlying mechanisms. Another important future research direction would be to study the link between behavior and circadian rhythms (cognitive responses, social influences…) that might be tightly intermingled. A good example could be the study of chronic desynchrony, for example in the context of circadian clock’s response to shiftworking, what has never been studied in the gray mouse lemur. Finally, special attention should be given to the description of sleep–wake cycles in adult and aged mouse lemurs, to confirm their intermediate characteristics between humans and rodents.

## Author Contributions

CH wrote the first draft of the manuscript. FP and MP wrote sections of the manuscript. All authors contributed to manuscript revision, read and approved the submitted version.

## Conflict of Interest Statement

The authors declare that the research was conducted in the absence of any commercial or financial relationships that could be construed as a potential conflict of interest.
