# Supplementary material for: The Biological Clock in Gray Mouse Lemur: Adaptive, Evolutionary and Aging Considerations in an Emerging Non-human Primate Model
Source: Front Physiol. 2019 Aug 9;10:1033. doi: 10.3389/fphys.2019.01033 (PMC6696974; doi:10.3389/fphys.2019.01033)
Supplement: Supplementary file 1 [file Table_1.DOCX]

|  | | **Mouse lemur**  **Supplementary material**  **Summary table of clock characteristics in gray mouse lemur and other mammalian species** | | | **Rodents** | | | **Human** | | | **Other primates** | | | |
| --- | --- | --- | --- | --- | --- | --- | --- | --- | --- | --- | --- | --- | --- | --- |
|  |  | *Ref* | *Adult* | *Age-related changes* | *Ref* | *Adult* | *Age-related changes* | *Ref* | *Adult* | *Age-related changes* | *Ref* | *Adult* | *Age-related changes* | |
| **Physiological parameters** | *Maximum Lifespan* | 1 | 10-12 years in captivity | | 2 | 5 years in captivity | |  | 100 years | | 3 | 35-40 years in captivity (*M. mulatta*) | | |
|  | *Core Temperature* | 4,5 | abrupt ↑ and ↓ in temperature at the onset and offset | ↓ of amplitude and hypothermia frequency | 6,7,  8,9 | abrupt ↑ and ↓ in temperature at the onset and offset | ↓ mean Tb, amplitude, phase advances, but dependent on "life style" | 10,11,  12,13,  14,15 | abrupt ↑ and ↓ in temperature at the onset and offset | ↓ amplitude and mean Tb, phase advance but dependent on "life style" | 16,17 | abrupt ↑ and ↓ in temperature at the onset and offset, Tb peaks delayed with the activity peaks (*E. albifrons*) | | ↓ amplitude and mean Tb, phase advances |
|  | *Melatonin levels* | 18 | progressive ↑ of melatonin, peak in the middle of the day | ↓ of diurnal pic of melatonin | 19,20, 21,22 | progressive ↑ of melatonin, peak in the middle of the night, long plateau before a brutal fall | ↓ of amplitude and level | 23,24, 25,26,  27 | progressive ↑ of melatonin during night, peak between 01:00 and 04:00 | ↓ of nocturnal peak and amplitude, phase advance | 16,28,  29 | progressive ↑ of melatonin during night, peak night values 2 to 15 fold higher than day values (*M. mulatta*) | | age-dependent decline in peak melatonin levels ( *M. mulatta*) |
|  | *Metabolism* | 30 | daily and seasonal fluctuations of metabolism rhythm in phase with LA and Tb rhythms | ↓ of the amplitude of the seasonal and daily variations of metabolism | 31,32,  33,34 | daily fluctuations of metabolism, coordinated expression of clock and clock-controlled genes in adipose tissues | shift to a sinusoidal cycle; ↓ of average and maximum rates of energy expenditure | 31,35 | [clock genes oscillate within human adipocytes, alterations in clock-genes correlated with obesity](http://science.sciencemag.org/content/330/6009/1349#ref-37) |  | 36,37 | daily fluctuations of oxygen consumption and heat production rhythms in phase with Tb and AL rhythms (*S. sciureus*) | |  |
|  | *Influence of nutrition* | 38,39 | deeper and longer hypothermias during chronic caloric restriction, ↓ of tau under resveratrol supplementation | ↓ of tau under resveratrol supplementation, but better synchronization | 40,41 | change of clock genes expression under resveratrol supplementation and high-fat diet; ↑ of tau, damped LA rhythm |  |  |  |  | 42 | maternal high-fat diet exposure in utero affects circadian gene expression (*M. fuscata*) | |  |
| **Daily Rhythms** | *Nocturnal/Diurnal* |  | nocturnal | | 43,44 | nocturnal (rat, mouse) nocturnal/diurnal (hamster) | |  | diurnal | |  | diurnal (*S. sciureus, M. mulatta*), nocturnal (*O. garnetii*, *A. trivirgatus*) | | |
|  | *Free-running period* | 45,46 | 23:00±0.03 | ↓ of tau | 47,48,  32,49,  19,20,  50,51 | 24.10 (hamster) ; 23.53 ± 0.08 (mouse) ; 24.20 ± 0.02 (rat) | ↓ of tau but ↑ in some lines (rat, mouse); depends (hamster) | 52,53 | 24.18 ± 0.04 | ↓ of tau or no variation | 16,54,  55,17  56 | 25.2±0.4h (*E. albifrons*); 23.8h (*A. trivirgatus*); 23.4 to 25.1h (*M. mulatta*) | | no variation (*M. mulatta*, *A. lemurinus*) |
|  | *Constant light exposure* | n.p. | inhibited LA, ↑ of tau, ↓ of melatonin levels |  | 57,58 | ↑ of tau with increasing light intensity (rat) |  | 59 | tau independent of light intensity |  | 60,61,  62, 63 | *↑of tau* and amplitude of Tb (*M. nemestrina*, *S.* *sciureus)* | |  |
|  | *Wake-sleep cycles* | 64,65 | fragmented sleep, slow frequency of SWS | ↓ activity during the active phase, more active wake, less slow wave sleep | 66,  67,  68, | fragmented sleep, high frequency of SWS | alteration of SWS, ↑ of desynchronized sleep; ↓ of NREM and REM sleep amplitude | 22,  69,  70,  71 | consolidated sleep of 8 hours, slow frequency of SWS | sleep fragmentation, ↓ of total sleep time, efficiency and SWS | 72,16 | consolidated sleep of 8 hours, slow frequency of SWS (*M. mulatta*) | | ↓ activity during the active phase, more active wake, less SWS |
|  | *Locomotor Activity* | 73,  4,8,  45 | daily oscillations | fragmentation of LA rhythms, advanced onset, ↓ of amplitude | 74,75,  76 | abrupt ↑ and ↓ in AL at the onset and offset | fragmentation and ↓ amplitude, phase advance; ↑ of interdaily variability | 22,77,78 | daily oscillations | fragmentation, phase advance; ↑ in intradaily variability, ↓ of amplitude | 16 | daily oscillations | | ↑ of interdaily variability (*M. mulatta*); ↓ of daily amplitude |
| **Photic Entrainment** | *Resynchronization after a phase delay or advance* | 79 | 2 or 3 days | identical | 80,81,  82 | 4 or 5 days | disrupted resynchronization of the liver Per1-driven luciferase after a 6h advance | 83,84 | questionable : 1 day or 3 to 4 days |  | 85,86,  87, 56 | 10 to 12 days (*O. garnetii*)  5.5 to 10 days (*A. lemurinus*) | | faster resynchronizati-on after a phase advance, no difference after a delay |
|  | *Influence of total cycle length* | n.p. | effective from 20h to 27h |  | 88 | effective from 19h to 32h (hamster) |  | 59 | effective from 22h to 27h |  | 89,56 | variable but in average effective from 22h to 28h | | no age effect (*A. lemurinus*) |
|  | *Influence of night length* | 4 | effective from 9h to 13h30 per day |  | 90 | effective from 3h to 18h per day (flying squirell, chipmunk) |  |  |  |  |  |  | |  |
|  | *Light sensitivity* | 91, 92 | photic entrainment threshold : 0.1 lux | ↓of light sensitivity | 93,94,  95,96,  97, 98,  99 | less than 0.01 lux (C57B1/6J mouse)  0.1 lux (rat) | ↓ of light sensitivity, ↓ of photic induction of early genes; ↓of shorter wavelength transmittance | 100 | 25 lux insufficient to entrain, 100 lux sufficient | ↓ of photic input to the clock | 91,  101 | photic entrainment threshold : 3-30 lux (*O. garnetii*) | |  |
|  | *PRC pattern* | 79 | fast resynchronization |  | 102  81  82 | similar pattern but slower resynchronization | tendency of reduced phase shifts | 83,  84 | fast resynchronization with lower amplitude | absence of age-related changes | 85,  86,  87 | fast resynchronization (owl and *S. sciureus*) | |  |
|  | *Response to chronic desynchrony* |  |  |  | 103,104,  105,106,  107, 108 | ↑of mortality, body weight, cardiovascular diseases | increased mortality compared to young individuals | 109,  110,  111, 112 | diabetes, cardiovascular and gastrointestinal diseases | ↓ of sleep efficiency |  |  | |  |
| **Behaviors** | *Social interactions influence* | 113 | synchronization of *tau* in free-run |  | 114,  115 | synchronization of LA and MR on the dominant animal rhythm |  | 116,  117 | reentrainment to a 8h delay through social cues, social entrainment to a 24h cycle in FR |  | 118,  119 | synchronization of *tau* in free-run (*C. jacchus*, *S. sciureus*) | |  |
|  | *Cognitive abilities* |  |  |  | 120 | Sleep-dependent memory dysfunction | ↓ amplitude of circadian activity associated with ↓ spatial long-term memory | 121,  122,  84 | clock effect on cognitive function in humans; alertness and performance dependent on circadian processes | ↓ of score to a cognitive task in older people after a forced desynchrony | 123, 124 | Altered cognitive performances in *M. mulatta* affected by circadian rhythm disorder | | daytime activity and sleep disorders correlated with memory performances |
| **Brain Molecular Aspects** | *Brain structure of circadian rhythms* | 125 | SCN |  | 126,  127,  128,  51,129 | SCN | ↓ in SCN rhythm amplitude, rhythms restoration, ↑ longevity after transplantation of a fœtal SCN | 130,  131 | SCN | neuronal degeneration of the SCN | 132, 133 | SCN | | ↓ of neuronal cells but ↑ of glial cells, no age-effect on SCN neuronal structure |
|  | *Bulbectomy influence* | 134 | alteration in circadian daily patterns |  | 135,  136, 137,  138 | alteration of tau, LA and Tb amplitude and level, reentrainment, *↓*of circadian cycling of c-Fos |  |  |  |  |  |  | |  |
|  | *AVP, VIP neurons* | 125  73 | daily oscillating rhythms, localization of AVP neurons in the SCN is dorsomedial, VIP is ventral | delayed peak of AVP and VIP neurons, no change of number or amplitude | 139,  140,  141,  142 | daily oscillating rhythms, localization of AVP neurons in the SCN is dorsomedial, VIP is ventral | ↓ in the number of AVP and VIP neurons in the SCN, loss of diurnal rhythmicity | 143,  144,  145,  146,  130,  147 | daily oscillating rhythms with a bimodal waveform, dorso-central localization of AVP neurons in the SCN, VIP is ventral | ↓ amplitude, volume and number of AVP neurons, ↓ in the number of male VIP neurons | 148 | daily oscillating rhythms, localization of AVP neurons in the SCN is central and dorsal, VIP is ventral (*S. apella*) | |  |
|  | *Calcium-binding protein calbindin* | 149 | daily oscillating rhythms, located in the ventral part of the SCN | ↓ daily variation in nuclear CalB immunoreactivity | 150 | located in the ventro-lateral part of the SCN |  | 151 | located in the ventral part of the SCN |  | 152 | located in the ventral part of the SCN | |  |

References:

1. Languille, S. *et al.* The grey mouse lemur: A non-human primate model for ageing studies. *Ageing Res. Rev.* **11**, 150–162 (2012).

2. Gorbunova, V., Bozzella, M. J. & Seluanov, A. Rodents for comparative aging studies: From mice to beavers. *Age (Omaha).* **30**, 111–119 (2008).

3. Bodkin, N. L., Alexander, T. M., Ortmeyer, H. K., Johnson, E. & Hansen, B. C. Mortality and Morbidity in Laboratory-maintained Rhesus Monkeys and Effects of Long-term Dietary Restriction. *Journals Gerontol. Ser. A Biol. Sci. Med. Sci.* **58**, B212–B219 (2003).

4. Perret, M. & Aujard, F. Daily hypothermia and torpor in a tropical primate: synchronization by 24-h light-dark cycle. *Am. J. Physiol. Regul. Integr. Comp. Physiol.* **281**, R1925–R1933 (2001).

5. Terrien, J., Zizzari, P., Epelbaum, J., Perret, M. & Aujard, F. Daily rhythms of core temperature and locomotor activity indicate different adaptive strategies to cold exposure in adult and aged mouse lemurs acclimated to a summer-like photoperiod. *Chronobiol. Int.* **26**, 838–853 (2009).

6. Refinetti, R., Ma, H. & Satinoff, E. Body temperature rhythms, cold tolerance, and fever in young and old rats of both genders. *Exp. Gerontol.* **25**, 533–543 (1990).

7. Halberg, J., Halberg, E., Regal, P. & Halberg, F. Changes with age characterize circadian rhythm in telemetered core temperature of stroke-prone rats. *Journals Gerontol.* **36**, 28–30 (1981).

8. Weinert, D. Circadian temperature variation and ageing. *Ageing Res. Rev.* **9**, 51–60 (2010).

9. Li, H. & Satinoff, E. Changes in circadian and sleep rhythms of body temperature in old rats. *Am. Physiol. Soc.* 208–214 (1995).

10. Gubin, D. G., Gubin, G. D., Waterhouse, J. & Weinert, D. The circadian body temperature rhythm in the elderly: Effect of single daily melatonin dosing. *Chronobiol. Int.* **23**, 639–658 (2006).

11. Vitiello, M. V. *et al.* Circadian temperature rhythms in young adult and aged men. *Neurobiol. Aging* **7**, 97–100 (1986).

12. Monk, T. H. Sleep and circadian rhythms. *Exp. Gerontol.* **26**, 233–243 (1991).

13. Monk, T., Buysse, D., Reynolds, C., Kupfer, D. & Houck, P. Circadian temperature rhythms of older people. *Exp. Gerontol.* **30**, 455–474 (1995).

14. Carrier, J., Paquet, J., Morettini, J. & Touchette, É. Phase advance of sleep and temperature circadian rhythms in the middle years of life in humans. *Neurosci. Lett.* **320**, 1–4 (2002).

15. Weinert, D. & Waterhouse, J. The circadian rhythm of core temperature: Effects of physical activity and aging. *Physiol. Behav.* **90**, 246–256 (2007).

16. Zhdanova, I. V. *et al.* Aging of intrinsic circadian rhythms and sleep in a diurnal nonhuman primate, Macaca mulatta. *J. Biol. Rhythms* **26**, 149–159 (2011).

17. Erkert, H. G. & Cramer, B. Chronobiological background to cathemerality: Circadian rhythms in Eulemur fulvus albifrons (Prosimii) and Aotus azarai boliviensis (Anthropoidea). *Folia Primatol.* **77**, 87–103 (2006).

18. Aujard, F. *et al.* Artificially accelerated aging by shortened photoperiod alters early gene expression (Fos) in the suprachiasmatic nucleus and sulfatoxymelatonin excretion in a small primate, Microcebus murinus. *Neuroscience* **105**, 403–412 (2001).

19. Mattam, U. & Jagota, A. Differential role of melatonin in restoration of age-induced alterations in daily rhythms of expression of various clock genes in suprachiasmatic nucleus of male Wistar rats. *Biogerontology* **15**, 257–268 (2014).

20. Manikonda, P. K. & Jagota, A. Melatonin administration differentially affects age-induced alterations in daily rhythms of lipid peroxidation and antioxidant enzymes in male rat liver. *Biogerontology* **13**, 511–524 (2012).

21. Reiter, R. J. The melatonin rhythm: both a clock and a calendar. *Experientia* **49**, 654–664 (1993).

22. Van Someren, E. J. W. Circadian and sleep disturbances in the elderly. *Exp. Gerontol.* **35**, 1229–1237 (2000).

23. Scholtens, R., Van Munster, B., Van Kempen, M. & De Rooij, S. Physiological melatonin levels in healthy older people : A systematic review. *J. Psychosom. Res.* **86**, 20–27 (2016).

24. Waldhauser, F., Kovacs, J. & Reiter, E. Age-related changes in melatonin livels in humans and its potential consequences for sleep disorders. *Experientia* **33**, 759–772 (1998).

25. Sharma, M. *et al.* Circadian rhythms of melatonin and cortisol in aging. *Biol. Psychiatry* **25**, 305–319 (1989).

26. Duffy, J. F. *et al.* Peak of circadian melatonin rhythm occurs later within the sleep of older subjects. *Am. J. Physiol. Endocrinal Metab.* **282**, 297–303 (2002).

27. Yoon, I., Kripke, D. F. & Elliott, J. A. Age-Related Changes of Circadian Rhythms and Sleep-Wake Cycles. *J. Am. Geriatr. Soc.* **51**, 1085–1091 (2003).

28. Stehle, J. H., Von Gall, C., Schomerus, C. & Korf, H. W. Of rodents and ungulates and melatonin: Creating a uniform code for darkness by different signaling mechanisms. *J. Biol. Rhythms* **16**, 312–325 (2001).

29. Reppert, S. M., Perlow, M. J., Tamarkin, L. & Klein, D. C. A Diurnal Melatonin Rhythm in Primate Cerebrospinal Fluid. *Endocrinology* **104**, 295–301 (2015).

30. Perret, M. & Aujard, F. Vieillissement et rythmes biologiques chez les primates. *Medecine/Sciences* **22**, 279–283 (2006).

31. Wu, X. *et al.* Induction of Circadian Gene Expression in Human Subcutaneous Adipose-derived Stem Cells. *Obesity* **15**, (2007).

32. Turek, F. W. *et al.* Obesity and Metabolic Syndrome in Circadian Clock Mutant Mice. *Science (80-. ).* **308**, 1043–1045 (2005).

33. Oishi, K. *et al.* Genome-wide Expression Analysis of Mouse Liver Reveals CLOCK-regulated Circadian Output Genes. *J. Biol. Chem.* **278**, 41519–41527 (2003).

34. Sacher, G. A. & Duffy, P. H. Age changes in rhythms of energy metabolism, activity and body temperature in Mus and Peromyscus. in *Aging and Biological Rhythms-H.V. Samis Jr et al. (eds)* 105–124 (1978).

35. Wu, X. *et al.* Expression Profile of mRNAs Encoding Core Circadian Regulatory Proteins in Human Subcutaneous Adipose Tissue: Correlation with Age and Body Mass Index. *Int. J. Obes.* **33**, 971–977 (2010).

36. Fuller, C. A., Sulzman, F. M. & Moore-Ede, M. C. Role of heat loss and heat production in generation of the circadian temperature rhythm of the squirrel monkey. *Physiol. Behav.* **34**, 543–546 (1985).

37. Robinson, E. L., Demaria-Pesce, V. H. & Fuller, C. A. Circadian rhythms of thermoregulation in the squirrel monkey (Saimiri sciureus). *Am. J. Physiol. Integr. Comp. Physiol.* **265**, 781–785 (1993).

38. Pifferi, F., Dal-Pan, A., Menaker, M. & Aujard, F. Resveratrol Dietary Supplementation Shortens the Free-Running Circadian Period and Decreases Body Temperature in a Prosimian Primate. *J. Biol. Rhythms* **26**, 271–275 (2011).

39. Pifferi, F., Dal-Pan, A., Languille, S. & Aujard, F. Effects of resveratrol on daily rhythms of locomotor activity and body temperature in young and aged grey mouse lemurs. *Oxid. Med. Cell. Longev.* **2013**, 1–7 (2013).

40. Oike, H. & Kobori, M. Resveratrol Regulates Circadian Clock Genes in Rat-1 Fibroblast Cells. *Biosci. Biotechnol. Biochem.* **72**, 3038–3040 (2008).

41. Kohsaka, A. *et al.* High-Fat Diet Disrupts Behavioral and Molecular Circadian Rhythms in Mice. *Cell Metab.* **6**, 414–421 (2007).

42. Suter, M. *et al.* Epigenomics: maternal high-fat diet exposure in utero disrupts peripheral circadian gene expression in nonhuman primates . *FASEB J.* **25**, 714–726 (2010).

43. Aschoff, J. Exogenous and endogenous components in circadian rhythms. *Cold Spring Harb. Symp. Quant. Biol.* **25**, 11–28 (1960).

44. Gattermann, R. *et al.* Golden hamsters are nocturnal in captivity but diurnal in nature. *Biol. Lett.* **4**, 253–255 (2008).

45. Cayetanot, F., Van Someren, E., Perret, M. & Aujard, F. Shortened seasonal photoperiodic cycles accelerate aging of the diurnal and circadian locomotor activity rhythms in a primate. *J. Biol. Rhythms* **20**, 461–469 (2005).

46. Aujard, F., Cayetanot, F., Terrien, J. & Van Someren, E. J. W. Attenuated effect of increased daylength on activity rhythm in the old mouse lemur, a non-human primate. *Exp. Gerontol.* **42**, 1079–1087 (2007).

47. McClung, C. *et al.* Regulation of dopaminergic transmission and cocaine reward by the Clockgene. *PNAS* **102**, 9377–9381 (2005).

48. Naylor, E. *et al.* The circadian clock mutation alters sleep homeostasis in the mouse. *J. Neurosci.* **20**, 8138–43 (2000).

49. Rudic, R. D. *et al.* BMAL1 and CLOCK, two essential components of the circadian clock, are involved in glucose homeostasis. *PLoS Biol.* **2**, (2004).

50. Kolker, D. E. *et al.* Aging Alters Circadian and Light-Induced Expression of Clock Genes in Golden Hamsters. *J. Biol. Rhythms* **18**, 159–169 (2003).

51. Nakamura, T. J. *et al.* Age-Related Decline in Circadian Output. *J. Neurosci.* **31**, 10201–10205 (2011).

52. Czeisler, C. A. *et al.* Stability , Precision , and Near – 24-Hour Period of the Human Circadian Pacemaker. *Science (80-. ).* **284**, 2177–2181 (1999).

53. Monk, T. H. Aging human circadian rhythms: Conventional wisdom may not always be right. *J. Biol. Rhythms* **20**, 366–374 (2005).

54. Masuda, K. & Zhdanova, I. V. Intrinsic activity rhythms in Macaca mulatta: Their entrainment to light and melatonin. *J. Biol. Rhythms* **25**, 361–371 (2010).

55. Fuller, C. A. & Edgar, D. M. Effects of Light Intensity on the Circadian Temperature and Feeding Rhythms in the Squirrel Monkey. *Physiol. Behav.* **36**, 687–691 (1986).

56. Rappold, I. & Erkert, H. G. Re‐entrainment, phase‐response and range of entrainment of circadian rhythms in Owl Monkeys (Aotus lemurinus g.) of different age. *Biol. Rhythm Res.* **25**, 133–152 (1994).

57. Coomans, C. P. *et al.* Detrimental effects of constant light exposure and high-fat diet on circadian energy metabolism and insulin sensitivity. *FASEB J.* **27**, 1721–1732 (2013).

58. Warren, W. S. & Cassone, V. M. The Pineal Gland: Photoreception and Coupling of Behavioral, Metabolic, and Cardiovascular Circadian Outputs. *J. Biol. Rhythms* **10**, 64–79 (1995).

59. Wever, R. A. Fractional Desynchronization of Human Circadian Rhythms : A Method for Evaluating Entrainment Limits and Functional Interdependencies Rfitger. *Eur. J. Physiol.* **396**, 128–137 (1983).

60. Aschoff, J. & Tokura, H. Circadian Activity Rhythms in Squirrel Monkeys: Entrainment by Temperature Cycles 1. *J. Biol. Rhythms* **1**, 91–99 (1986).

61. Takasu, N., Nigi, H. & Tokura, H. Effects of diurnal bright/dim light intensity on circadian core temperature and activity rhythms in the Japanese macaque. *Jpn.J Physiol* **52**, 573–578 (2002).

62. Sulzman, F. M., Fuller, C. A. & Moore-Ede, M. C. Tonic effects of light on the circadian system of the squirrel monkey. *J. Comp. Physiol.* **129**, 43–50 (1979).

63. Tokura, H. & Aschoff, J. Circadian activity rhythms of the pig-tailed macaque, Macaca nemestrina, under constant illumination. *Eur. J. Physiol.* **376**, 241–243 (1978).

64. Pifferi, F. *et al.* Sleep changes during aging: electroencephalography (EEG) study in a non-human primate. Neuroscience Congress Washington, DC (2011).

65. Pifferi, F. *et al.* Effects of dietary resveratrol on the sleep-wake cycle in the non-human primate gray mouse lemur (Microcebus murinus). *Chronobiol. Int.* **29**, 261–270 (2012).

66. Revel, F. G., Gottowik, J., Gatti, S., Wettstein, J. G. & Moreau, J. L. Rodent models of insomnia: A review of experimental procedures that induce sleep disturbances. *Neurosci. Biobehav. Rev.* **33**, 874–899 (2009).

67. Panagiotou, M., Vyazovskiy, V. V., Meijer, J. H. & Deboer, T. Differences in electroencephalographic non-rapid-eye movement sleep slow-wave characteristics between young and old mice. *Sci. Rep.* **7**, 1–12 (2017).

68. Welsh, D. K., Richardson, G. S. & Dement, W. C. Effect of age on the circadian pattern of sleep and wakefulness in the mouse. *J. Gerontol.* **41**, 579–586 (1986).

69. McCarley, R. W. Mechanisms and models of REM sleep control. *Archives Italiennes de Biologie* **142**, 429–467 (2004).

70. Luca, G. *et al.* Age and gender variations of sleep in subjects without sleep disorders. *Ann. Med.* **47**, 482–491 (2015).

71. Crowley, K. Sleep and sleep disorders in older adults. *Neuropsychol. Rev.* **21**, 41–53 (2011).

72. Hsieh, K.-C., Robinson, E. L. & Fuller, C. A. Sleep architecture in unrestrained Rhesus monkeys (Macaca mulatta) synchronized to 24-hour light-dark cycles. *Sleep* **31**, 1239–1250 (2008).

73. Aujard, F., Cayetanot, F., Bentivoglio, M. & Perret, M. Age-related effects on the biological clock and its behavioral output in a primate. *Chronobiol. Int.* **23**, 451–460 (2006).

74. Weinert, H., Weinert, D. & Sturm, J. Age-dependant changes in the stability of the daily activity rhythms of laboratory mice. *Int. J. Mamm. Biol.* **65**, 21–32 (2000).

75. Scarbrough, K., Losee-Olson, S., Wallen, E. P. & Turek, F. W. Aging and photoperiod affect entrainment and quantitative aspects of locomotor behavior in Syrian hamsters. *Am. J. Physiol. Integr. Comp. Physiol.* **272**, 1219–1225 (1997).

76. van Gool, W. A., Witting, W. & Mirmiran, M. Age-related changes in circadian sleep-wakefulness rhythms in male rats isolated from time cues. *Brain Res.* **413**, 384–387 (1987).

77. Huang, Y. L. *et al.* Age-associated difference in circadian sleep-wake and rest-activity rhythms. *Physiol. Behav.* **76**, 597–603 (2002).

78. Van Someren, E. J. W. & Riemersma-Van Der Lek, R. F. Live to the rhythm, slave to the rhythm. *Sleep Med. Rev.* **11**, 465–484 (2007).

79. Schilling, A., Richard, J. P. & Servière, J. Effect of aging on circadian activity in gray mouse lemurs. *Int. J. Primatol.* **22**, 25–42 (2001).

80. Pittendrigh, C. & Daan, S. A functional analysis of circadian pacemakers in nocturnal rodents. I. The Stability and Lability of Spontaneous Frequency. *J. Comp. Physiol.* **106**, 291–331 (1976).

81. Pohl, H. Difference in responses of the circadian system to light in the Syrian hamster. *Physiol. Zool.* **57**, 509–520 (1984).

82. Benloucif, S., Masana, M. I. & Dubocovich, M. L. Light-induced phase shifts of circadian activity rhythms and immediate early gene expression in the suprachiasmatic nucleus are attenuated in old C3H/HeN mice. *Brain Res.* **747**, 34–42 (1997).

83. St Hilaire, M. A. *et al.* Human phase response curve to a 1h pulse of bright white light. *J. Physiol.* **590**, 3035–3045 (2012).

84. Duffy, J. F., Dijk, D.-J., Klerman, E. B. & Czeisler, C. A. Later endogenous circadian temperature nadir relative to an earlier wake time in older people. *Am. J. Physiol. Integr. Comp. Physiol.* **275**, 1478–1487 (1998).

85. Rauth-Widmann, B., Thiemann-Jäger, A. & Erkert, H. G. Significance of nonparametric light effects in entrainment of circadian rhythms in owl monkeys (aotus lemurinus griseimembra) by light-dark cycles. *Chronobiol. Int.* **8**, 251–266 (1991).

86. Wechselberger, E. & Erkert, H. G. Characteristics of the light-induced phase-response of circadian activity rhythms in common marmosets, Callithrixj. jacchus (Primates - Cebidae). *Chronobiol. Int.* **11**, 275–284 (1994).

87. Hoban, T. M. & Sulzman, F. M. Light effects on circadian timing system of a diurnal primate, the squirrel monkey. *Am. J. Physiol. Integr. Comp. Physiol.* **249**, 274–280 (1985).

88. Chiesa, J. J., Díez-Noguera, A. & Cambras, T. Effects of transient and continuous wheel running activity on the upper and lower limits of entrainment to light-dark cycles in female hamsters. *Chronobiol. Int.* **24**, 215–234 (2007).

89. Härter, L. & Erkert, H. G. Alteration of circadian period length does not influence the ovarian cycle length in common marmosets, Callithrix j. jacchus (primates). *Chronobiol. Int.* **10**, 165–175 (1993).

90. DeCoursey, P. J. LD ratios and the entrainment of circadian activity in a nocturnal and a diurnal rodent. *J. Comp. Physiol.* **78**, 221–235 (1972).

91. Erkert, H. G., Gburek, V. & Scheideler, A. Photic entrainment and masking of prosimian circadian rhythms (Otolemur garnettii, Primates). *Physiol. Behav.* **88**, 39–46 (2006).

92. Gomez, D., Barbosa, A., Théry, M., Aujard, F. & Perret, M. Age Affects Photoentrainment in a Nocturnal Primate. *J. Biol. Rhythms* **XX**, 1–8 (2012).

93. Asai, M. *et al.* Circadian profile of Per gene mRNA expression in the suprachiasmatic nucleus, paraventricular nucleus, and pineal body of aged rats. *J. Neurosci. Res.* **66**, 1133–1139 (2001).

94. Sutin, E. L., Dement, W. C., Heller, H. C. & Kilduff, T. S. Light-induced gene expression in the suprachiasmatic nucleus of young and aging rats. *Neurobiol. Aging* **14**, 441–446 (1993).

95. Lupi, D., Semo, M. & Foster, R. G. Impact of age and retinal degeneration on the light input to circadian brain structures. *Neurobiol. Aging* **33**, 383–392 (2012).

96. Zhang, Y. *et al.* Effects of aging on light-induced phase-shifting of circadian behavioral rhythms, fos expression and Creb phosphorylation in the Hamster suprachiasmatic nucleus. *Neuroscience* **70**, 951–961 (1996).

97. Zhang, Y. *et al.* Effects of aging on lens transmittance and retinal input to the suprachmasmatic nucleus in golden hamsters. *Neurosci. Lett.* **258**, 167–170 (1998).

98. Ebihara, S. & Tsuji, K. Entrainment of the circadian activity rhythm to the light cycle: Effective light intensity for a Zeitgeber in the retinal degenerate C3H mouse and the normal C57BL mouse. *Physiol. Behav.* **24**, 523–527 (1980).

99. Ruis, J. F., Rietveld, W. J. & Buys, J. P. Properties of parametric photic entrainment of circadian rhythms in the rat. *Physiol. Behav.* **50**, 1233–1239 (1991).

100. Gronfier, C., Wright, K. P., Kronauer, R. E. & Czeisler, C. A. Entrainment of the human circadian pacemaker to longer-than-24-h days. *Proc. Natl. Acad. Sci.* **104**, 9081–9086 (2007).

101. Erkert, H. G. Diurnality and nocturnality in nonhuman primates: Comparative chronobiological studies in laboratory and nature. *Biol. Rhythm Res.* **39**, 229–267 (2008).

102. Pittendrigh, C. & Daan, S. A functional analysis of circadian pacemakers in nocturnal rodents. II. The Variability of Phase Response Curves. *J. Comp. Physiol.* **106**, 333–355 (1976).

103. Davidson, A. *et al.* Chronic Jet-Lag Increases Mortality in Aged Mice. *Curr. Biol.* **16**, 914–916 (2006).

104. Nelson, W. & Halberg, F. Schedule-shifts, circadian rhythms and lifespan of freely-feeding and meal-fed mice. *Physiol. Behav.* **38**, 781–788 (1986).

105. Tsai, L. L. & Tsai, Y. C. The effect of scheduled forced wheel activity on body weight in male F344 rats undergoing chronic circadian desynchronization. *Int. J. Obes.* **31**, 1368–1377 (2007).

106. Penev, P., Kolker, D., Zee, P. & Turek, F. Chronic circadian desynchronization decreases the survival of animals with cardiomyopathic heart disease. *Am. Physiol. Soc.* **22**, 200–206 (2002).

107. Hurd, M. W. & Ralph, M. The significance of circadian organization for longevity in the golden hamster. *J. Biol. Rhythms* **13**, 430–436 (1998).

108. Preuss, F. *et al.* Adverse effects of chronic circadian desynchronization in animals in a “challenging” environment. *Am. J. Physiol. Integr. Comp. Physiol.* **295**, 2034–2040 (2008).

109. Costa, G. Shift work and occupational medicine: an overview. *Occup. Med. (Chic. Ill).* **53**, 83–88 (2003).

110. Haus, E. & Smolensky, M. Biological clocks and shift work: Circadian dysregulation and potential long-term effects. *Cancer Causes Control* **17**, 489–500 (2006).

111. Knutsson, A. Health disorders of shift workers. *Occup. Med. (Chic. Ill).* **53**, 103–108 (2003).

112. Dijk, D. J., Duffy, J. F., Kiel, E., Shanahan, T. L. & Czeisler, C. A. Ageing and the circadian and homeostatic regulation of human sleep during forced desynchrony of rest, melatonin and temperature rhythms. *J. Physiol.* **516**, 611–627 (1999).

113. Séguy, M. Facteurs d’environnement, physiques et sociaux, et maintien de l’homeostasie chez un primate tropical [PhD thesis]. in *University Pierre et Marie Curie (Paris)* (2005).

114. Crowley, M. & Bovet, J. Social synchronization of circadian rhythms in deer mice (Peromyscus maniculatus). *Behav. Ecol. Sociobiol.* **7**, 99–105 (1980).

115. Stupfel, M. *et al.* Light-dark and societal synchronization of respiratory and motor activities in laboratory mice, rats, guinea-pigs and quails. *Comp. Biochem. Physiol.* **70**, 265–274 (1981).

116. Honma, S. *et al.* Differential effects of bright light and social cues on reentrainment of human rhythms. *Am. Physiol. Soc.* 528–535 (1995).

117. J . Aschoff , M . Fatranská , H . Giedke , P . Doerr, D. . S. and H. . Human Circadian Rhythms in Continuous Darkness : Entrainment by Social Cues. *Science (80-. ).* **171**, 213–215 (1971).

118. Erkert, H. G. & Schardt, U. Social Entrainment of Circadian Activity Rhythms in Common Marmosets, Callithrix-J-Jacchus (Primates). *Ethology* **87**, 189–202 (1991).

119. Rajaratnam, S. M. W. & Redman, J. R. Social contact synchronizes free-running activity rhythms of diurnal palm squirrels. *Physiol. Behav.* **66**, 21–26 (1999).

120. George, O. *et al.* Low brain allopregnanolone levels mediate flattened circadian activity associated with memory impairments in aged rats. *Biol. Psychiatry* **68**, 956–963 (2010).

121. wyatt1999.pdf.

122. Dijk, D.-J. ‐J, DUFFY, J. F., Czeisler, C. & A. Circadian and sleep/wake dependent aspects of subjective alertness and cognitive performance. *J. Sleep Res.* **1**, 112–117 (1992).

123. Zhdanova, I. V. *et al.* Familial circadian rhythm disorder in the diurnal primate, Macaca mulatta. *PLoS One* **7**, 1–14 (2012).

124. Haley, G. E. *et al.* Circadian activity associated with spatial learning and memory in aging rhesus monkeys. *Exp. Neurol.* **217**, 55–62 (2009).

125. Cayetanot, F., Bentivoglio, M. & Aujard, F. Arginine-vasopressin and vasointestinal polypeptide rhythms in the suprachiasmatic nucleus of the mouse lemur reveal aging-related alterations of circadian pacemaker neurons in a non-human primate. *Eur. J. Neurosci.* **22**, 902–910 (2005).

126. Davidson, A. J., Yamazaki, S., Arble, D. M., Menaker, M. & Block, G. D. Resetting of central and peripheral circadian oscillators in aged rats. *Neurobiol. Aging* **29**, 471–477 (2008).

127. Cai, A., Scarbrough, K., Hinkle, D. A. & Wise, P. M. Fetal grafts containing suprachiasmatic nuclei restore the diurnal rhythm of CRH and POMC mRNA in aging rats. *Am. J. Physiol.* **273**, R1764-70 (1997).

128. Viswanathan, N. & Davis, F. C. Suprachiasmatic nucleus grafts restore circadian function in aged hamsters. *Brain Res.* **686**, 10–16 (1995).

129. Li, H. & Satinoff, E. Fetal tissue containing the suprachiasmatic nucleus restores multiple circadian rhythms in old rats. *Am. J. Physiol. Integr. Comp. Physiol.* **275**, 1735–1744 (1998).

130. Hofman, M. A. the Human Circadian Clock and Aging. *Chronobiol. Int.* **17**, 245–259 (2000).

131. Swaab, D. F., Fliers, E. & Partiman, T. S. The suprachiasmatic nucleus of the human brain in relation to sex, age and senile dementia. *Brain Res.* **342**, 37–44 (1985).

132. Engelberth, R. C. G. J. *et al.* Morphological Changes in the Suprachiasmatic Nucleus of Aging Female Marmosets (Callithrix jacchus). *Biomed Res. Int.* **2014**, 1–10 (2014).

133. Roberts, D. E., Killiany, R. J. & Rosene, D. L. Neuron numbers in the hypothalamus of the normal aging rhesus monkey: Stability across the adult lifespan and between the sexes. *J. Comp. Neurol.* **520**, 1181–1197 (2012).

134. Perret, M., Aujard, F., Séguy, M. & Schilling, A. Olfactory bulbectomy modifies photic entrainment and circadian rhythms of body temperature and locomotor activity in a nocturnal primate. *J. Biol. Rhythms* **18**, 392–401 (2003).

135. Marcilhac, A. *et al.* Effects of bilateral olfactory bulbectomy on circadian rhythms of ACTH, corticosterone, motor activity and body temperature in male rats. *Arch. Physiol. Biochem.* **105**, 552–559 (1997).

136. Vinkers, C. H. *et al.* Olfactory bulbectomy induces rapid and stable changes in basal and stress-induced locomotor activity, heart rate and body temperature responses in the home cage. *Neuroscience* **159**, 39–46 (2009).

137. Giardina, W. J. & Redek, R. J. Effects of imipramine on the nocturnal behavior of bilateral olfactory bulbectomized rats. *Biol. Psychiatry* **29**, 1200–1208 (1991).

138. Forster, C., Parkes, J. & Cox, B. Effects of olfactory bulbectomy and peripherallyinduced anosmia on thermoregulation in the rat: susceptibility to antidepressant type drugs. *J. Pharm. Pharmacol.* **32**, 630–634 (1980).

139. Roozendaal, B., van Gool, W. A., Swaab, D. F., Hoogendijk, J. E. & Mirmiran, M. Changes in vasopressin cells of the rat suprachiasmatic nucleus with aging. *Brain Res.* **409**, 259–264 (1987).

140. Kalló, I., Kalamatianos, T., Piggins, H. D. & Coen, C. W. Ageing and the diurnal expression of mRNAs for vasoactive intestinal peptide and for the VPAC2 and PAC1 receptors in the suprachiasmatic nucleus of male rats. *J. Neuroendocrinol.* **16**, 758–766 (2004).

141. Kawakami, F. *et al.* Loss of day-night differences in VIP mRNA levels in the suprachiasmatic nucleus of aged rats. *Neurosci. Lett.* **222**, 99–102 (1997).

142. Chee, C. A., Roozendaal, B., Swaab, D. F., Goudsmit, E. & Mirmiran, M. Vasoactive intestinal polypeptide neuron changes in the senile rat suprachiasmatic nucleus. *Neurobiol. Aging* **9**, 307–312 (1988).

143. Kalsbeek, A., Fliers, E., Hofman, M. A., Swaab, D. F. & Buijs, R. M. Vasopressin and the Output of the Hypothalamic Biological Clock Neuroendocrinology. *J. Neuroendocrinol.* **22**, 362–372 (2010).

144. Ishunina, T. A. & Swaab, D. Vasopressin and Oxytocin Neurons of the Human Supraoptic and Paraventricular Nucleus; Size Changes in Relation to Age and Sex. *J. Clin. Endocrinol. Metab.* **84**, 4637–4644 (1999).

145. Hofman, M. A. & Swaab, D. F. Diurnal and Seasonal Rhythms of Neuronal Activity in the Suprachiasmatic Nucleus of Humans. *J. Biol. Rhythms* **8**, 283–295 (1993).

146. Hofman, M. A. & Swaab, D. F. Alterations in circadian rhythmicity of the vasopressin-producing neurons of the human suprachiasmatic nucleus (SCN) with aging. *Brain Res.* **651**, 134–142 (1994).

147. Witting, W., Mirmiran, M., Bos, N. P. A. & Swaab, D. F. Effect of light intensity on diurnal sleep-wake distribution in young and old rats. *Brain Res. Bull.* **30**, 157–162 (1993).

148. Campos, L. M. G., Cruz-rizzolo, R. J., Watanabe, I., Pinato, L. & Nogueira, M. I. Efferent projections of the suprachiasmatic nucleus based on the distribution of vasoactive intestinal peptide (VIP) and arginine vasopressin (AVP) immunoreactive fibers in the hypothalamus of Sapajus apella. *J. Chem. Neuroanat.* **57**–**58**, 42–53 (2014).

149. Cayetanot, F., Nygård, M., Perret, M., Kristensson, K. & Aujard, F. Plasma levels of interferon-γ correlate with age-related disturbances of circadian rhythms and survival in a non-human primate. *Chronobiol. Int.* **26**, 1587–1601 (2009).

150. Arvanitogiannis, A., Robinson, B., Beaulé, C. & Amir, S. Calbindin-D28K immunoreactivity in the suprachiasmatic nucleus and the circadian response to constant light in the rat. *Neuroscience* **99**, 397–401 (2000).

151. Mai, J. K., Teckhaus, L. & Sofroniew, M. V. Evidence for Subdivisions in the Human Suprachiasmatic Nucleus. *J. Comp. Neurol.* **305**, 508–525 (1991).

152. Cavalcante, J. S. *et al.* Calcium-binding proteins in the circadian centers of the common marmoset (Callithrix jacchus) and the rock cavy (Kerodon rupestris) brains. *Brain Res. Bull.* **76**, 354–360 (2008).
